# Characterization of the tyrosine-hydroxylase immunoreactive components of the basal subpallium in sharks—toward an identification of a basal subpallial complex

**DOI:** 10.3389/fnana.2025.1620527

**Published:** 2025-08-26

**Authors:** Isabel Rodríguez-Moldes, Catalina Sueiro, Iván Carrera, Idoia Quintana-Urzainqui, Eva Candal

**Affiliations:** Neurodevo Group, Aquatic One Health Research Center (ARCUS), Department of Functional Biology (Cell Biology Section), Faculty of Biology, University of Santiago de Compostela, Santiago de Compostela, Spain

**Keywords:** chondrichthyans, *Scyliorhinus canicula*, area superficialis basalis, basal ganglia, pallidum, striatum, amygdala, forebrain evolution

## Abstract

Comparative studies on the forebrain across different model organisms are necessary to investigate the origin and degree of evolutionary conservation of this brain region and its derivatives. The catshark *Scyliorhinus canicula* has become a reliable model representative of cartilaginous fishes (Chondrichthyans), the oldest divergent lineage of extant gnathostomes (jawed vertebrates). Previous studies on the chemoarchitecture, connectivity, and development of the subpallium of *S. canicula* revealed the existence of subdivisions with an embryological origin and genetic specification similar to those of tetrapods, supporting homology with their basal ganglia and certain amygdaloid components. To better understand the evolutionary origin of these subpallial components, we present here a summary of the main neuroanatomical, chemoarchitectural, and developmental features of the *area superficialis basalis* of *S. canicula*, a nuclear aggrupation of the basal forebrain of all Chondrichthyans that has been related to the basal ganglia and the amygdala. Particular emphasis has been placed on characterizing the tyrosine hydroxylase-positive components of the subpallium to discern their involvement in the structural organization of putative basal ganglia homologs in the catshark. We propose a new interpretation that considers the *area superficialis basalis* as the central part of a subpallial complex formed also by its neighboring territories, where the basic components of the basal ganglia and the amygdala of gnathostomes would be integrated.

## 1 Introduction

The Chondrichthyans (cartilaginous fishes) form the sister group of the Osteichthyans, which includes the bony fishes and the tetrapods, and thus represent the earliest divergent lineage of the extant gnathostomes (jawed vertebrates). This strategic phylogenetic position makes cartilaginous fishes a key group for understanding vertebrate evolution. Comparisons between Chondrichthyans and Osteichthyans are crucial for identifying conserved traits that may help to outline the ancestral state of any specific character in the gnathostome group.

Similar to tetrapods, the telencephalon in Chondrichthyans develops through evagination—a morphogenetic process that involves the enlargement of the central lumen of the anterior neural tube to form the telencephalic ventricles, followed by an outward expansion of the telencephalic walls. The telencephalon of Chondrichthyans, as in other vertebrates, consists of two main histogenetic subdivisions, the pallium (topographically dorsal) and the subpallium (topographically ventral). The evaginated telencephalon (which forms the telencephalic hemispheres) includes the pallium and part of the subpallium, which in turn contains the basal ganglia (divided into striatal and pallidal regions) and parts of the amygdala. The non-evaginated telencephalon (which forms the impar telencephalon or telencephalon medium, that is, the portion of the telencephalon that encloses an unpaired ventricle) consists of a single subpallial component, the preoptic area (POA).

Two remarkable facts that characterize the telencephalon of all Chondrichthyans studied so far: the presence of abundant dopaminergic cells (dopamine- and tyrosine hydroxylase-immunoreactive; Meredith and Smeets, 1987; Northcutt et al., 1988; Stuesse et al., 1990, 1994; Carrera et al., 2012; Quintana-Urzainqui et al., 2012) in the pallium (particularly abundant in the dorsal pallium), and the existence of the *area superficialis basalis,* the most conspicuous structure in the subpallium characterized by a laminar organization of densely packed neurons. The presence of pallial dopaminergic neurons is shared with mammals (Smeets and González, 2000; Benavides-Piccione and DeFelipe, 2007; Björklund and Dunnett, 2007), lungfishes (López and González, 2017), and Chondrosteans (Adrio et al., 2002). In contrast, a structure similar to the *area superficialis basalis* has not been reported in the subpallium of any other vertebrate.

The *area superficialis basalis* has been described as a corticoid cell layer (Manso and Anadón, 1993), as a U-shaped layer of small cells (Smeets et al., 1983), or as a plate of densely packed bipolar neurons (Northcutt et al., 1988) parallel to the ventral and ventrolateral surface of the subpallium. Different names have been used to refer to this nuclear aggrupation based on the functions proposed for it, about which there is no consensus. Due to its suggested role as an olfactory fiber receptor, it was referred to as *nucleus postolfactorius*, *cortex olfactoria*, and olfactory tubercle/*tuberculum olfactorium* (revised in Schroeder and Ebbesson, 1974; Smeets et al., 1983). However, experimental investigations showed that only the lateralmost portion of this nuclear aggrupation receives secondary olfactory projections (Ebbesson and Heimer, 1970; Smeets et al., 1983). Other classic names were *nucleus taeniae*, which suggests homology with the amygdala, and hypostriatum, in reference to its topographical relation to a supposedly adjacent striatal territory. In the following, we will use its most common name, *area superficialis basalis*, following Johnston (1911).

This intriguing nuclear aggrupation has been proposed to be homologous to the basal amygdala of land vertebrates by Northcutt (1978) on the basis that the *area superficialis basalis* receives massive input from the olfactory-dominated lateral pallium as shown by Ebbesson (1972). However, because of its neurochemical characteristics, it has been considered as a possible homolog to the globus pallidus (dorsal pallidum) of tetrapods (Northcutt et al., 1988) and, alternatively, based on histochemical characteristics and topography, as homologous to the ventral striatum (Northcutt et al., 1988). The possibility that the *area superficialis basalis* contains neurons homologous to those in both the globus pallidus and olfactory tubercle of amniotes has also been considered (Northcutt et al., 1988).

To clarify the nature of this subpallial structure and its potential homology with pallial or subpallial territories defined in tetrapods, we have compiled our previous data and critically reviewed the available literature concerning the cytoarchitectural organization, connections, chemoarchitecture, and developmental origin of the *area superficialis basalis* and neighboring territories in the catshark *Scyliorhinus canicula*, one of the main chondrichthyan model species (Coolen et al., 2008; Mayeur et al., 2024).

Taking all of this information into account, we propose a new interpretation that considers the *area superficialis basalis* and its surrounding territories a subpallial complex, which integrates structures homologous to the basic components of both the amygdala and basal ganglia in tetrapods.

## 2 Materials and methods

### 2.1 Experimental animals and tissue preparation

Male and female adults (45–60 cm in length), juveniles (9–12 cm in length), and early prehatching embryos at stage 32 proposed by Ballard et al. (1993) of the catshark *S. canicula* L. were used in this study. This embryonic stage is relevant to the present study because it marks the beginning of the second half of the catshark embryonic development and, more importantly, because its brain has reached a relatively mature organization, and the subpallial neuronal groups that we have characterized in juveniles and adults begin to differentiate and become recognizable.

Animals were supplied by local fishermen (adults), the Marine Biological Model Supply Service of the Center National de la Recherche Scientifique (CNRS) and Université Pierre et Marie Curie (UPMC) Roscoff Biological Station (France), and Aquaria of O Grove (Pontevedra, Spain) and Finisterrae (A Coruña, Spain). Adequate measures were taken to minimize animal pain or discomfort. The original research reported herein was performed according to the regulations and laws established by the European Union (2010/63/UE) and by the Spanish Royal Decree 1386/2018 for the care and handling of animals in research and was approved by the Ethics Committee of the University of Santiago de Compostela. Animals were deeply anesthetized with 0.5% MS-222 in seawater prior to any experimental procedure.

Animals were intracardially perfused with elasmobranch Ringer’s solution followed by 4% paraformaldehyde (PFA) in elasmobranch’s phosphate buffer (containing urea), and the brains were immediately dissected out and immersed in the same fixative for 4 h. After being rinsed in phosphate-buffered saline (PBS), the brain and embryos were cryoprotected with 30% sucrose in PBS, embedded in OCT compound (Tissue Tek, Torrance, CA, United States), and frozen with liquid-nitrogen-cooled isopentane. Parallel series of sections (12–20 μm thick) were cut on a cryostat in sagittal, horizontal, or transverse planes in relation to the longitudinal body axis and mounted on Superfrost Plus (Menzel-Glasser, Madison, WI, United States) slides. For more details about the procedure, see the study by Carrera et al. (2012). For general cytoarchitectonic analysis, some serial sections were stained with hematoxylin–eosin.

### 2.2 Immunohistochemistry

As primary antisera, we used monoclonal mouse anti-tyrosine hydroxylase (anti-TH, Millipore, Billerica, MA, United States; 1:500–1,000) and polyclonal rabbit anti-glutamic acid decarboxylase (anti-GAD, Chemicon, Temecula, CA, United States; dilution 1:1,000); anti-Met-Enkephalin (anti-MetEnk, Affiniti, Exeter, United Kingdom; dilution 1:1,000); anti-substance P (anti-SP, Chemicon®, Merck KGaA, Darmstadt, Germany; 1:1,000); anti-calretinin (anti-CR, Swant, Bellinzona, Switzerland; 1:250–500); anti-doublecortin (anti-DCX, Cell Signaling Technology, Beverly, MA, United States; dilution 1:300); anti-serotonin (anti-5HT DiaSorin/Immunostar, Hudson, WI, United States; 1,2,500–5,000); anti-calbindin D-28 k (anti-CB Swant, Bellinzona, Switzerland; 1:800); and anti-Pax6 (Covance, Emeryville, CA, United States; 1:400).

Details of the procedures and the specificity of the antibodies used have been published elsewhere (TH: Carrera et al., 2012; GAD: Sueiro et al., 2004; anti-Met-Enkephalin: Rodríguez-Díaz et al., 2011; Substance P: Rodríguez-Moldes et al., 1993; CR, DCX: Pose-Méndez et al., 2014; 5HT: Carrera et al., 2008b; CB: Sánchez-Farías and Candal, 2015; Pax6: Ferreiro-Galve et al., 2012). Briefly, sections were pre-treated with 0.01 M citrate buffer (pH 6.0) for 30 min at 95°C for heat-induced epitope retrieval, rinsed in 0.05 M Tris-buffered saline (TBS; pH 7.4), and incubated overnight with the primary antibody in a humid chamber at room temperature (RT). After rinsing in TBS, appropriate secondary antibodies (horseradish peroxidase [HRP]-conjugated goat anti-mouse and anti-rabbit, Bio-Rad, Hercules, California, United States; diluted 1:200) were incubated for 1 h at RT. After rinsing with TBS, the immunolabeling was visualized with 0.25 mg/mL diaminobenzidine tetrahydrochloride (DAB; Merck/Millipore Sigma, Burlington, Massachusetts, United States) in TBS with 0.00075% H_2_O_2_ and 2.5 mg/mL nickel ammonium sulfate (blue precipitate) or with SIGMAFAST™ 3,30-DAB tablets (Merck; brown precipitate). All dilutions were made with TBS containing 2% bovine serum albumin (Merck), 15% normal goat serum (Dako, now part of Agilent, Santa Clara, CA, United States) or normal donkey serum (Agilent), and 0.2% Triton X-100 (Merck). Finally, the sections were dehydrated, mounted, and coverslipped.

As tyrosine hydroxylase is the rate-limiting enzyme in the synthesis of catecholamines (dopamine, adrenaline, and noradrenaline), TH antibodies do not allow for the identification of the type of amine synthesized. However, they provide a valuable method for studying the distribution of most catecholamine-synthesizing neurons (Smeets and González, 2000). The TH antibody employed in this study displays wide species cross-reactivity (revised in Carrera et al., 2012) and has been mostly used to decipher catecholaminergic systems in the catshark central nervous system (Molist et al., 1993; Carrera et al., 2005, 2012; Sueiro et al., 2003, 2007; Candal et al., 2008; Sobrido-Cameán et al., 2020; Rodríguez-Moldes et al., 2022). In these studies, it has been widely assumed that the majority of cells labeled with the TH antibody are catecholamine-synthesizing cells, and that most of them are dopaminergic. However, considering that the presence of the TH enzyme at immunohistochemically detectable levels is not always a reliable condition for identifying dopaminergic neurons (Björklund and Dunnett, 2007) and that this antibody appears to reveal only the catecholamine-synthesizing neurons immunoreactive to the isoform TH1 (Filippi et al., 2010; Yamamoto et al., 2010), here we will refer to neurons and fibers showing immunoreactivity to the antibody we used as TH-immunoreactive (ir) or TH-positive.

### 2.3 *In situ* hybridization on sections

Sense and antisense digoxigenin-UTP-labeled Dlx2, Nkx2.1, and Lhx9 (*ScDlx2*, *ScNkx2.1*, and *ScLhx9*) were synthesized directly by transcription *in vitro*. *In situ* hybridization was performed on cryostat sections of embryos at stage 32 following standard protocols (Coolen et al., 2007). For information about the origin of the probes, see the study by Quintana-Urzainqui et al. (2015). These probes were selected from a collection of *S. canicula* embryonic complementary DNA (cDNA) library, constructed in pSPORT1, and submitted to high-throughput EST sequencing. Selected cDNA fragments were cloned into plasmid vectors, namely, pSPORT vectors. Sense and antisense digoxigenin-UTP-labeled and fluorescein-UTP-labeled probes were synthesized directly by *in vitro* transcription using linearized recombinant plasmid DNA or cDNA fragments prepared by polymerase chain reaction (PCR) amplification of the recombinant plasmids as templates. Briefly, sections were permeabilized with proteinase K, hybridized with sense or antisense probes overnight at 65°C, and incubated with alkaline phosphatase-coupled anti-digoxigenin and anti-fluorescein antibodies (1,2,000, Roche Applied Science, Mannheim, Germany) overnight at 4°C. The color reaction was performed in the presence of BM-Purple (Roche). Control sense probes did not produce any detectable signal.

### 2.4 Image acquisition

Bright-field images were obtained with an Olympus BX51 photomicroscope (Olympus, now Evident) equipped with an Olympus DP71 color digital camera.

## 3 Results

### 3.1 Cytoarchitectural organization of the basal subpallium in the catshark

**Figure 1:**
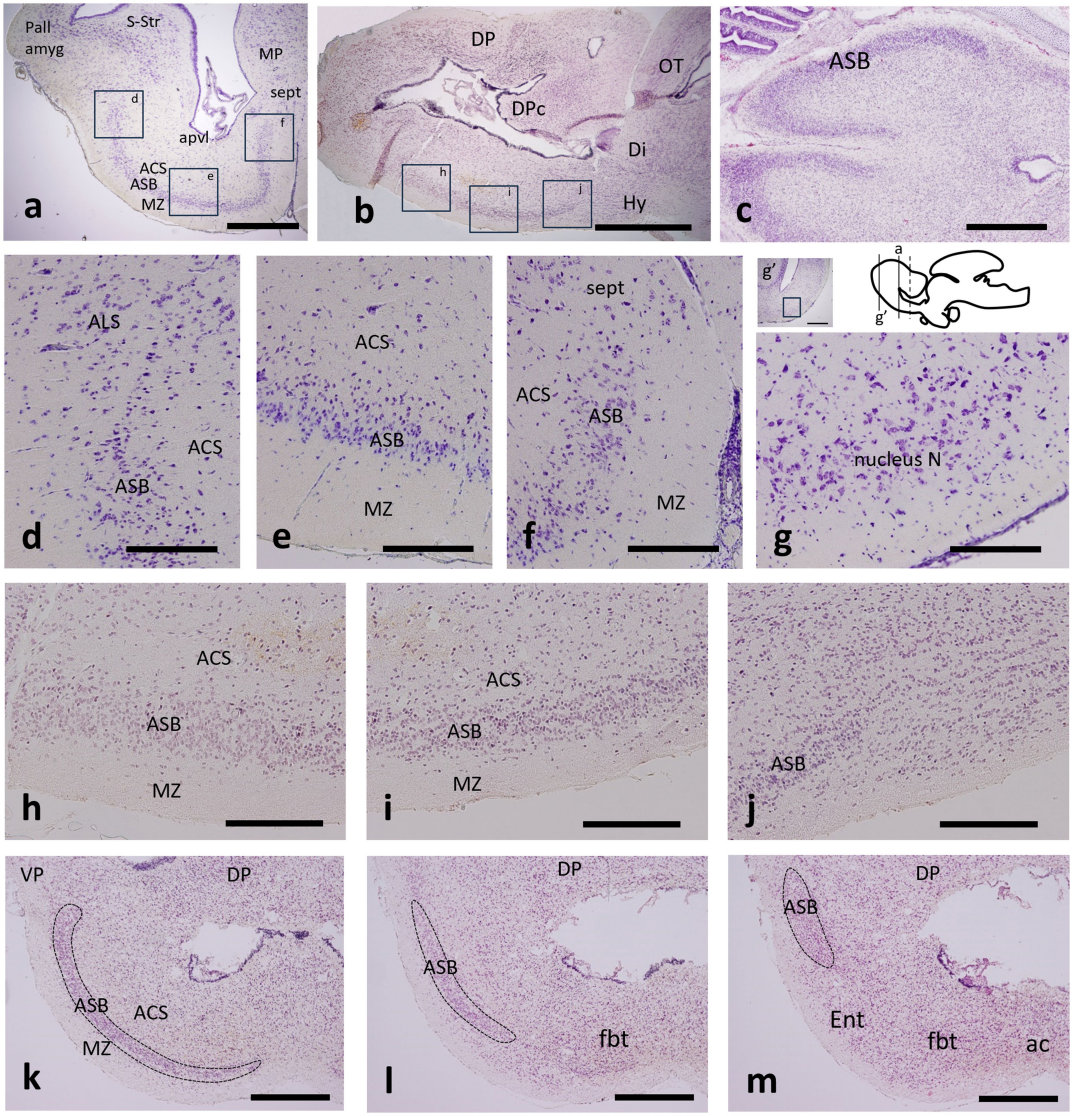
Transverse **(a,d–g,k–m)**, sagittal **(b,h–j)** and horizontal **(c)** sections through the telencephalon of catshark adult **(a,d–g)**, juvenile **(b,h–j)** and stage-32 embryo **(c,k–m)** stained with cresyl violet **(a,d–g)** and hematoxylin–eosin **(b,c,h–m)** to show the *area superficialis basalis* (ASB) and neighboring territories. The approximate correspondence of figures d–f and h–j is indicated in boxed areas in figures a and b. **(g)** Detail of the nucleus N at the area boxed in the inset of a section more rostral than that shown in figure a, as indicated by solid bars on the thumbnail scheme of a sagittal view of the adult brain; the dotted bar indicates the approximate level of the entopeduncular nucleus referred to in Figure 3h **(k–m)** Sequential transverse sections through the transition from evaginated **(k)** to non-evaginated **(m)** subpallium to show the ASB (dotted territory) ending caudally in a laterodorsal position. Scale bar: 2 mm **(a,b,g’)**, 1 mm **(c,k–m)**, and 500 μm **(d–j)**. For abbreviations, see the “Abbreviations” list.

**Figure 2:**
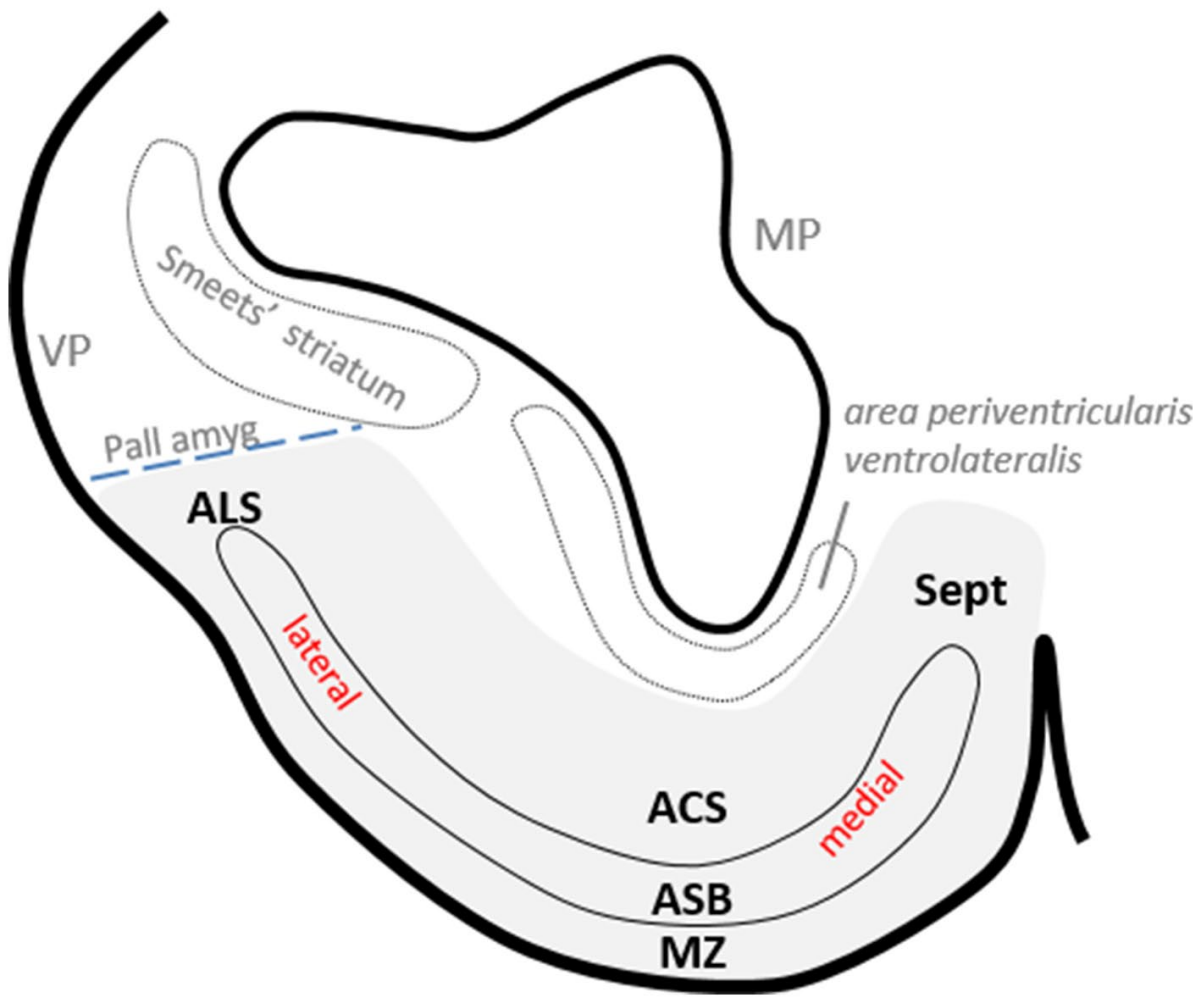
Drawing of a transverse section of the catshark subpallium at the level shown in Figure 1a to show the relative position of *area superficialis basalis* (ASB) and neighboring territories in the basal subpallium (gray-shaded area) and with respect to certain pallial areas. The dotted line indicates the presumed pallial–subpallial boundary.

The subpallium of the catshark presents an evident radial dimension when considering its main structure, the *area superficialis basalis*, in relation to its adjacent outer and inner territories, that is, the marginal neuropile and the *area centralis subpaliallis*, which represents the intermediate area between the *area superficialis basalis*, and the periventricular stratum occupied by the *area periventricularis lateralis* (Figures 1a, 2).

The *area superficialis basalis* is clearly distinguished from the neighboring territories because its densely packed cells form a stratum that occupies most of the outer part of the subpallium, as can be seen in transverse, sagittal or horizontal sections of catshark adults, juveniles and embryos at stage 32 stained with hematoxylin–eosin or with cresyl violet (Figures 1a–c). Its high cell density allows it to be distinguished by contrast even in contrast tomography sections, when none of its elements are stained (Rodríguez-Moldes et al., 2022; see also Figures 5g,h in the study by Mayeur et al., 2024). It is the largest cell group of the subpallium and, along most of its length, it extends lateromedially from levels close to the lateroventral pallium to levels close to the median septum (Figures 1d–f). Rostrally (topographically), the *area superficialis basalis* borders the nucleus N, which also occupies the external stratum and extends lateromedially, but is distinguished by its lower cell density (Figure 1g). The *area superficialis basalis* extends throughout the entire basal region of the telencephalon (Figures 1h–j). Along most of its length, it forms a broad stratum extended lateromedially, but caudally (topographically), such lateromedial extension becomes progressively reduced until it occupies an exclusively lateral position at the impar telencephalon (Figures 1k–m). At this level, the *area superficialis basalis* borders the entopeduncular nucleus, which occupies a mediolateral position here, but lacks the compact cellular organization of the *area superficialis basalis* (Figure 1m).

In a previous study, we identified a cell population in the catshark subpallium that caps laterally the *area superficialis basalis* and is limited by the pallial amygdala at the ventral pallium (Rodríguez-Moldes et al., 2022). Since it had not been named, in this study, we refer to it as the *area lateralis subpallialis* (Figures 1d, 2).

### 3.2 Chemoarchitecture of the developed basal subpallium

#### 3.2.1 Tyrosine hydroxylase immunoreactivity

**Figure 3:**
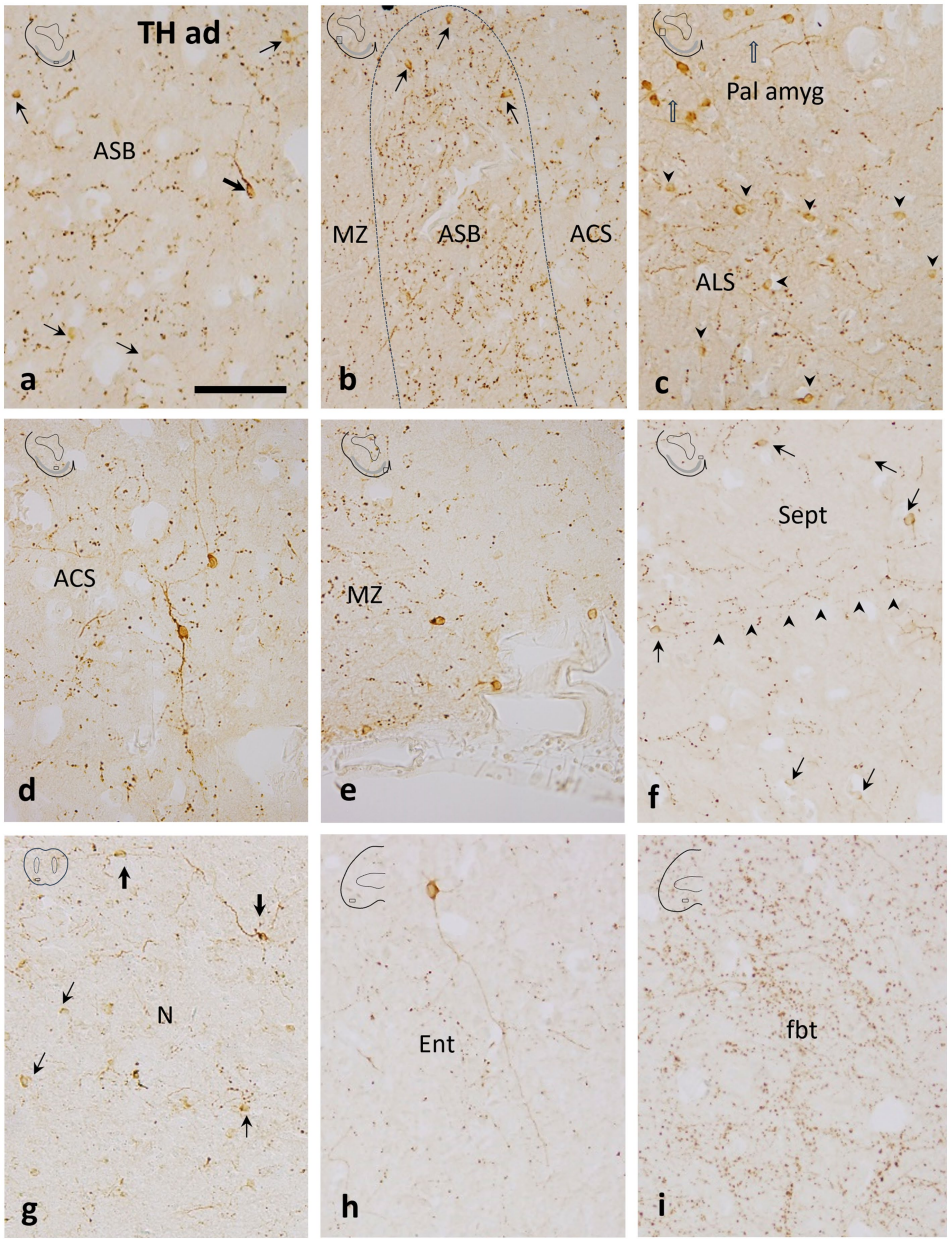
Details of transverse sections through the adult subpallium of the catshark at the areas indicated in boxes in thumbnail schemes to show tyrosine hydroxylase immunoreactivity in the *area superficialis basalis* (ASB) and surrounding territories. **(a)** Central part of the ASB with scarce small round (thin arrows) and some larger bipolar (thick arrow) TH-ir cells. Note the moderate TH-ir innervation, mostly formed by TH-ir boutons; **(b)** Mid-lateral part of the ASB with scarce small round TH-ir cells (thin arrows). Compare to a to note that the TH-ir innervation is denser than in the central part of the ASB; **(c)** Section just dorsal to b to show small round TH-ir cells grouped in the *area lateralis subpallialis* (ALS; arrowheads) and more dorsally, the larger TH-ir cells of the pallial amygdala (Pall amyg; open arrows); **(d)** A large bipolar TH-ir cell in the *area centralis subpallialis* (ACS). Note the relatively long TH-ir processes and the moderate density of TH-ir boutons; **(e)** Marginal neuropile (MZ) at a ventromedial level (median line is at the right) showing some small TH-ir cells lining the external surface; **(f)** Medial subpallial area just dorsal to figure e showing abundant small TH-ir cells in the septal region (Sept). Note the medio-lateral orientation of most varicose fibers (arrowheads); **(g)** Nucleus N with small, faint TH-ir cells (thin arrows) and larger TH-ir cells (thick arrows) with long processes; **(h)** Large TH-ir cell with a long process in the entopeduncular nucleus (Ent). Note the less dense TH-ir innervation compared with the ASB; **(i)** Dense TH-ir innervation in the *fasciculus basalis telencephali* (fbt). Scale bar: 200 μm.

In the catshark, scarce small TH-immunoreactive (ir) cells were described as homogeneously distributed at different levels of the *area superficialis basalis* (Carrera et al., 2012; Quintana-Urzainqui et al., 2012). Here we show that at least two types of TH-ir cells can be distinguished: small, round, and weakly labeled, more numerous cells, and slightly larger and sparser bipolar cells with long immunoreactive dendrites without a preferential orientation (Figures 3a,b).

TH-ir cells have been observed in all the territories adjacent to the *area superficialis basalis*, although differences can be noted among them. A dense population of small round TH-ir cells was observed at the *area lateralis subpallialis* (Figure 3c), capping the *area superficialis basalis* (see also Rodríguez-Moldes et al., 2022). This subpallial group differs from the more dorsal TH-ir population of cells located at pallial levels (Figure 3c; see also Figures 2i,j of Rodríguez-Moldes et al., 2022), which has been presumed to be the ventral pallial component of the catshark amygdalar complex, equivalent to the lateral amygdala of amphibians (Rodríguez-Moldes et al., 2022).

In the *area centralis subpallialis*, round and faintly stained TH-ir cells were also observed together with the large bipolar TH-ir cells with intensely labeled long dendrites, which appear to be more abundant than those in the *area superficialis basalis* (Figure 3d). Monopolar TH-ir cells were seen in the periphery of the marginal neuropile of the *area superficialis basalis*, some of them placed close to blood vessels (Figure 3e). Relatively abundant small TH-ir cells were seen in the septal region (Figure 3f). Similarly to the *area superficialis basalis*, small weakly labeled TH-ir cells and scarcer large TH-ir cells with long immunoreactive processes were seen in its rostral continuation, the nucleus N (Figure 3g). Just caudally to the *area superficialis basalis,* weak labeled TH-ir cells were also seen scattered in the entopeduncular nucleus. However, intensely labeled cells with long radial TH-ir dendrites were occasionally seen (Figure 3h).

In juvenile catsharks, the *area superficialis basalis* has been recognized as one of the subpallial regions with the densest TH-ir innervation, together with the *area periventricularis ventrolateralis* and the striatum of Smeets (Carrera et al., 2012). Here, we show in adult catsharks that beaded fibers, boutons and, to a lesser extent, long, thin dendritic processes characterize the conspicuous TH-ir innervation of the *area superficialis basalis* (Figures 3a,b). The TH-positive innervation in the *area superficialis basalis* is denser, especially at lateral levels, than in adjacent territories such as the *area centralis subpallialis*, marginal neuropile, and *area lateralis subpallialis*, and septal area, which have moderate to scarce TH-ir innervation (Figures 3a–f). Comparatively, the nuclei adjacent rostrally and caudally to the *area superficialis basalis* (nucleus N and entopeduncular nucleus, respectively) present the least dense subpallial TH-ir innervation (Figures 3g,h).

Abundant coarse TH-ir fibers coursed through the *fasciculus basalis telencephali* or basal forebrain bundle (Figure 3i), which has been defined as the main communication channel between the subpallium and the more caudal brain regions, consisting mainly of fibers originating from or projected to the striatum and *area superficialis basalis* (Smeets et al., 1983).

#### 3.2.2 Glutamic acid decarboxylase immunoreactivity (GABAergic marker)

**Figure 4:**
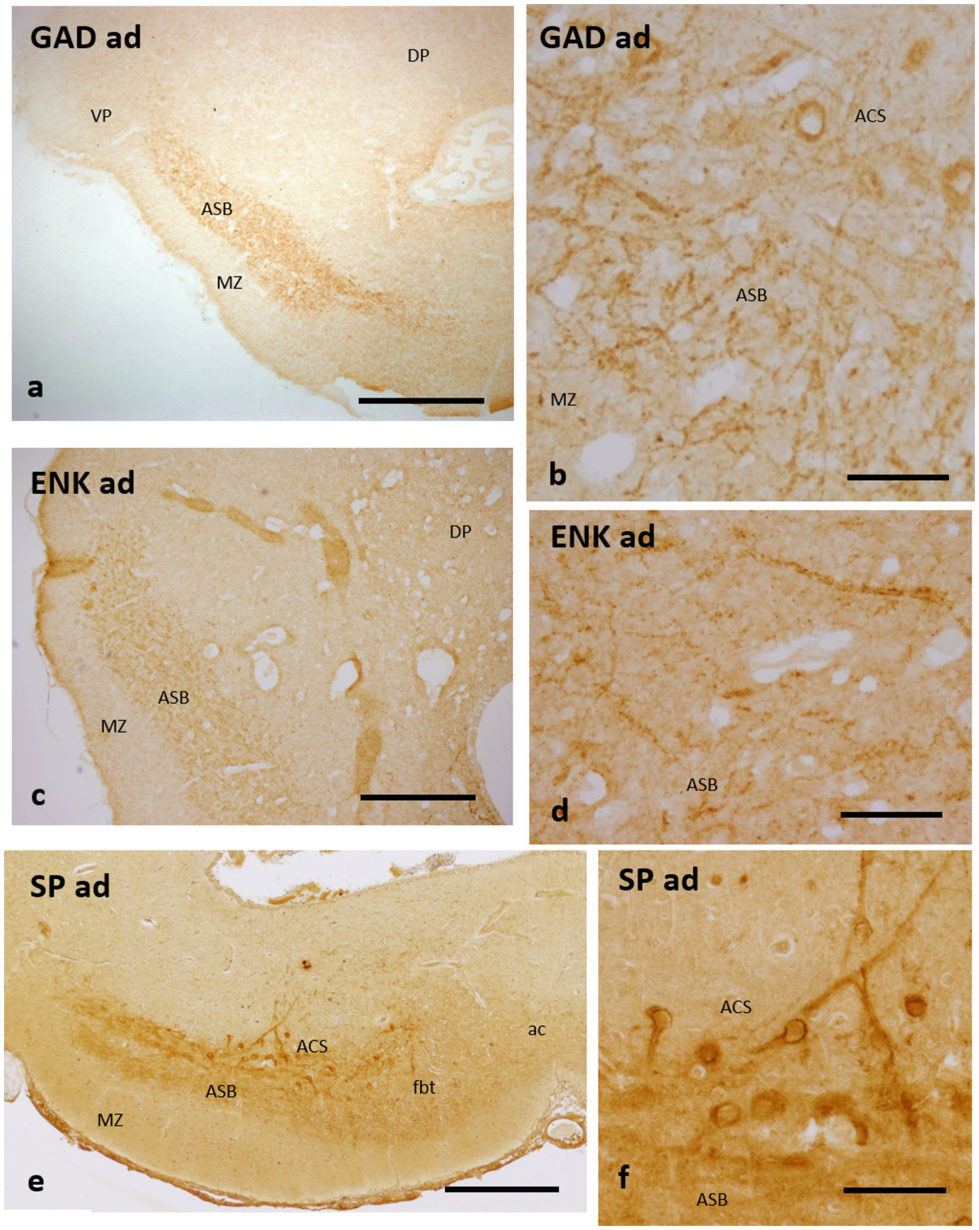
Transverse sections of the catshark subpallium of adults at the transition between the evaginated and non-evaginated (impar) telencephalon showing immunoreactivity to GAD **(a,b)**, Met-Enkephalin **(c,d)** and Substance P **(e,f)** in the caudal part of the *area superficialis basalis* and *area centralis subpallialis.* **(b,d,f)** Details to show perisomatic and peridendritic immunoreactive boutons to the three markers on immunonegative cells. For abbreviations, see the “Abbreviations” list. Scale bar: 2 mm **(a)**; 1 mm **(c,e)**; and 200 μm **(b,d,f)**.

It has been pointed out that the *area superficialis basalis* of catshark presents scarce GAD-ir cells and a dense gamma-aminobutyric acid (GABAergic) innervation (Quintana-Urzainqui et al., 2012), which contrasts with the scant to moderate GABAergic innervation at the different subpallial regions. Here, we show a regionalization in the distribution of GABAergic structures in relation to *area superficialis basalis* and its neighboring territories. At levels of the evaginated subpallium, the GABAergic innervation is higher at the marginal neuropile and *area centralis subpallialis* than within the proper *area superficialis basalis* (not shown). But at levels of the impar telencephalon (the territory derived from the non-evaginated subpallium), the highest GABAergic innervation is found at the proper *area superficialis basalis* and in the *area centralis subpallialis* (Figures 4a,b). As shown above (Figures 1k–m), in this subpallial territory, the extent of the *area superficialis basalis* shifts from lateromedial to only lateral at caudal levels. At its caudal pole, the *area superficialis basalis* is wedge-shaped, with its narrowest part apparently overlapping with pallial territories (Figures 1k–m, 4a). At this level, most of the GABAergic innervation is formed by abundant GAD-ir terminals covering thick dendrites, probably belonging to the large bipolar cells of the *area superficialis basalis*, and immunonegative somata mainly located in the *area centralis subpallialis* (Figure 4b).

#### 3.2.3 Enkephalin and substance P immunoreactivities

Scarce ENK-ir cells but a rich ENK-ir innervation were described in the *area superficialis basalis* of the catshark (Quintana-Urzainqui et al., 2012). Such ENK-ir innervation characteristically presents abundant ENK-ir terminals surrounding immunonegative somata and thick dendrites, which, as noted for the GABAergic innervation (see above), are especially dense at the caudal levels and at the interface with the *area centralis subpallialis* (Figures 4c,d).

Some small SP-ir neurons, as well as abundant SP-ir fibers and their terminal fields, have been described surrounding immunonegative cells and fibers in the *area superficialis basalis* of the catshark (Rodríguez-Moldes et al., 1993; Quintana-Urzainqui et al., 2012). A similar pattern of SP immunoreactivity was observed by these authors in the *area centralis subpallialis*, with major immunoreactivity bordering immunonegative somata and dendritic processes, in addition to SP-ir cells. Here we show that, similar to that observed with GAD and ENK antibodies, the highest density of perisomatic and peridendritic SP-ir boutons is observed at caudal levels of the *area superficialis basalis* and its interface with the *area centralis subpallialis* (Figures 4e,f).

#### 3.2.4 Calretinin immunoreactivity

**Figure 5:**
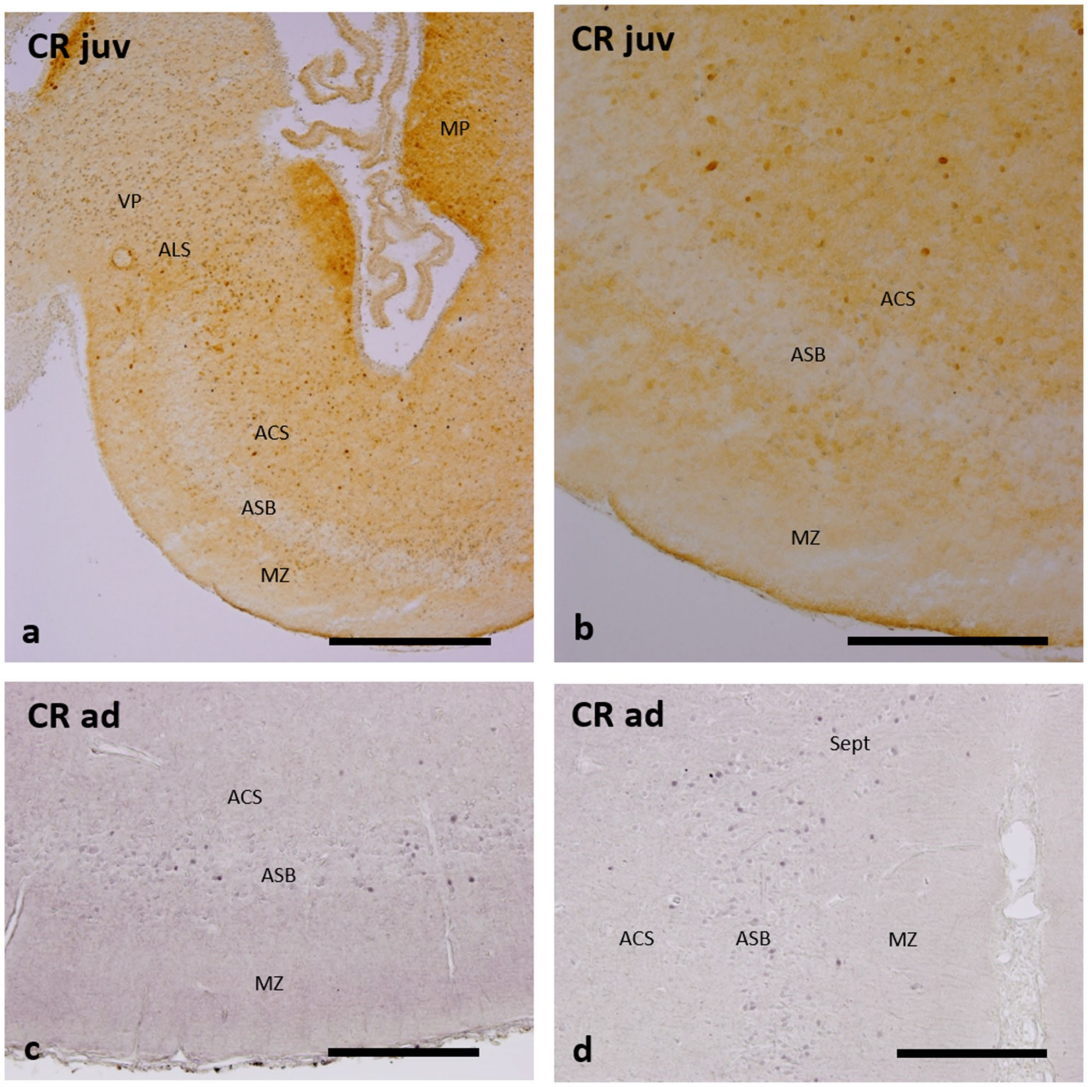
Transverse and sections of the catshark subpallium of juveniles **(a,b)** and adults **(c,d)**, showing immunoreactivity to calretinin (CR). For abbreviations, see the “Abbreviations” list, and for identification of the regions, see Figure 2. Scale bar: 1 mm **(a)** and 500 μm **(b–d)**.

The distribution of CR-ir cells in the subpallium of catshark adults and juveniles (Figures 5a–d) is rather similar to that of CB-ir cells described in adults (Rodríguez-Moldes et al., 1990) and in juveniles (Rodríguez-Moldes et al., 2022), with CR-ir cells in the *area superficialis basalis*, *area lateralis subpallialis*, *area centralis subpallialis*, and marginal neuropile. The main distinction with the CB immunoreactivity is the lesser abundance of CR-ir cells within the *area superficialis basalis,* especially in juveniles (Figures 5a,b), and the scarcity of these cells in adjacent territories (Figures 5a–d), except for the *area lateralis subpallialis* (Figure 5a) and septal region (Figure 5d), which exhibit a moderate density of CR-ir cells.

### 3.3 Cyto-, chemo-, and genoarchitecture of early cell populations of the basal subpallium

**Figure 6:**
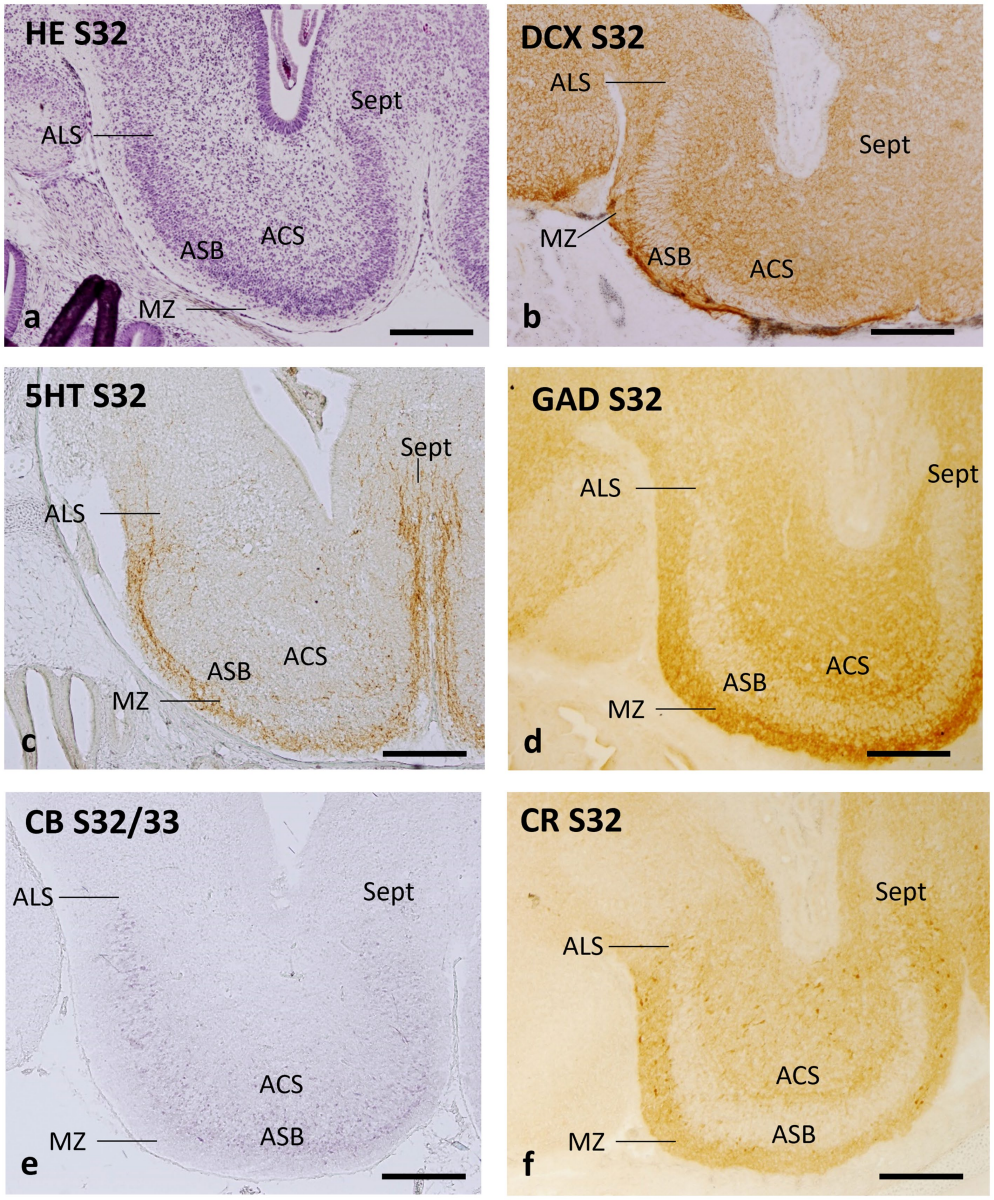
Transverse sections of the catshark subpallium of embryos at stage 32 stained with hematoxylin–eosin (HE; **a**) and immunohistochemistry to doublecortin (DCX; **b**), serotonin (5HT, **c**), GAD **(d)**, calbindin (CB; **e**), and calretinin (CR; **f**). For abbreviations, see the “Abbreviations” list. Scale bar: 500 μm.

At stage 32, the characteristic cytoarchitecture of the *area superficialis basalis* is clearly recognized with hematoxylin–eosin and with different immunomarkers (Figure 6). In contrast, at stage 31, no signs of differentiation of the nuclear cell groups are observed, as can be seen in the sections of these embryos labeled with different markers in previous studies (Quintana-Urzainqui et al., 2012, 2015; Rodríguez-Moldes et al., 2017). In fact, at stage 31, the standard primary layers of the telencephalic walls are formed and the topological organization of most neuronal systems is established (Quintana-Urzainqui et al., 2012). Still, it is at stage 32 when the mature basic structure of the telencephalon is progressively achieved and the cytoarchitecture and organization of the telencephalon become largely similar to that of juveniles and adults (Rodríguez-Moldes et al., 2022). Therefore, we have selected embryonic stage 32 to define the early organization of the *area superficialis basalis* and neighboring territories.

Hematoxylin–eosin-stained transverse sections of early stage 32 demonstrate that the region with the highest cell density in the subpallium at this stage of development is an external stratum that can be unequivocally identified as the *area superficialis basalis* (Figure 6a). The neighboring territories can be virtually identified as *area lateralis subpallialis*, *area centralis subpallialis*, marginal neuropile, and septal area (Figure 6a).

The antibody against DCX, a microtubule-associated protein related to neurogenesis, migration of immature neurons and synaptogenesis, also allows unequivocal identification of the *area superficialis basalis* due to radial organization of the fine immunoreactive structures that traverse it (probably growing fibers and/or fields of synaptogenesis), which contrasts with the adjacent territories (Figure 6b).

Pioneer telencephalic 5HT-ir fibers have been described reaching the telencephalon through the dorsolateral subpallial walls in catshark embryos at stage 31 (Carrera et al., 2008b). This area, associated with the pallial–subpallial boundary, continues to have a high density of 5HT-ir fibers as the embryo develops, as seen at stage 32, where 5HT-ir fibers also innervate different subpallial regions (Figure 6c). Interestingly, the poor 5HT-ir innervation of the recognizable *area superficialis basalis* contrasts with the high density of 5HT-ir fibers bordering it externally, in the territory corresponding to the marginal neuropile (Figure 6c). The *area centralis subpallialis*, *area lateralis subpallialis,* and septal region present scarce to moderate 5HT-ir innervation (Figure 6c).

The distribution of GAD immunoreactivity in the subpallium at stage 32 is essentially similar to that of juveniles and adults, with poor GAD immunoreactivity at the *area superficialis basalis*, which contrasts with the dense GAD-ir innervation at the nearby territories (Figure 6d).

In embryos at stage 32, CB-ir cells are abundant in the *area superficialis basalis* and moderately dense in adjacent territories, such as the *area centralis subpallialis*, *area lateralis subpallialis*, and septum (Figure 6e).

The distribution pattern of CR-ir cells in stage-32 embryos appears basically similar to that of CB-ir cells, but the density of CR-ir cells within the *area superficialis basalis* is considerably lower (Figure 6f; see also Figure 5E of Rodríguez-Moldes et al., 2022, and Figures 1l,m of Ferreiro-Galve et al., 2008).

**Figure 7:**
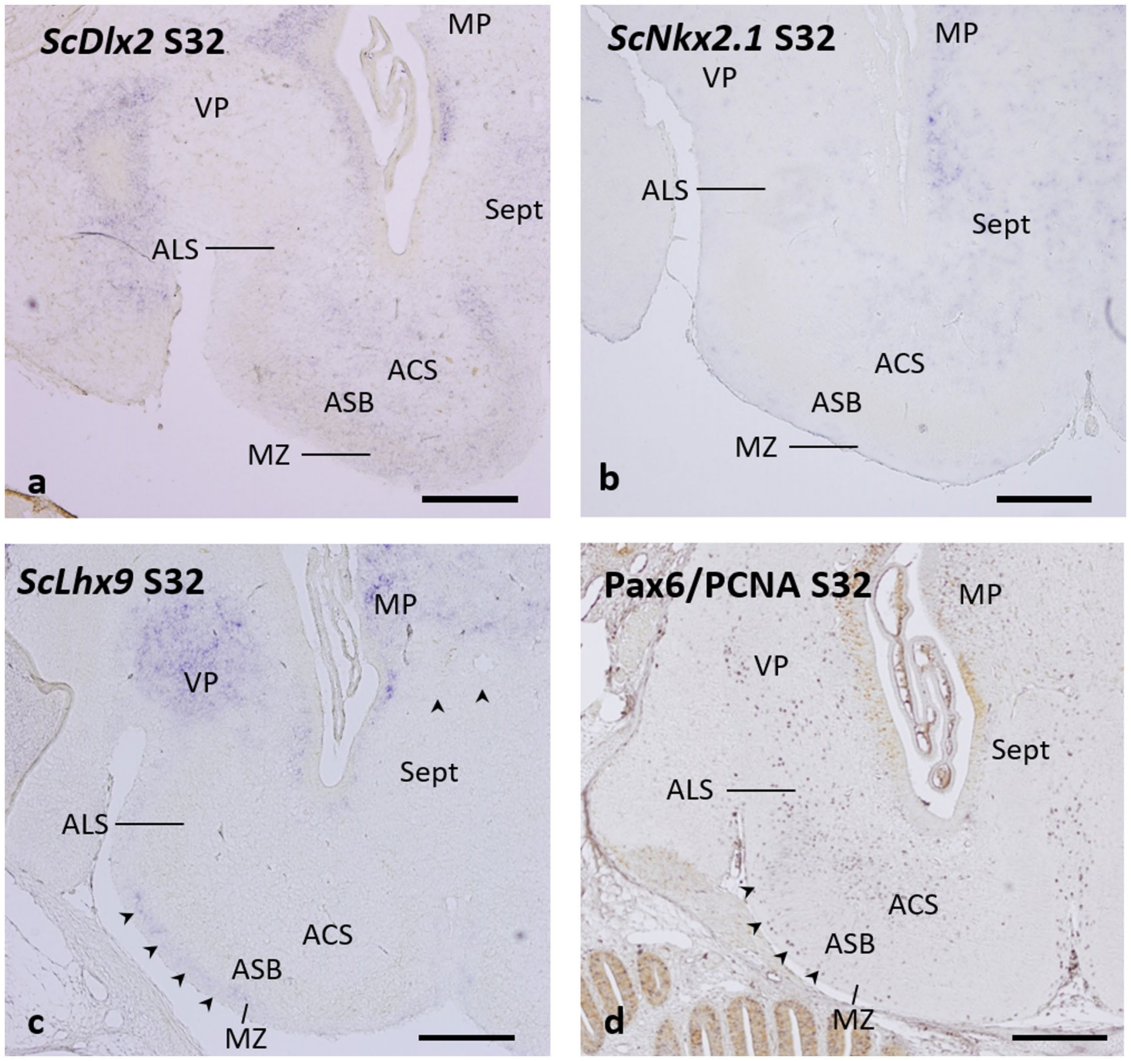
Transverse sections of the catshark subpallium of embryos at stage 32 showing the expression of *ScDlx2* **(a)**, *ScNkx2.1* **(b)**, and *ScLhx9* **(c)**, and the distribution of immunoreactivity to Pax6 (purple) and PCNA (brown) **(d)**. Arrowheads in c and d point to streams of *ScLhx9*-expressing cells (in **c**) and of Pax6-ir cells (in **d**) occupying the marginal neuropile. For abbreviations, see the “Abbreviations” list. Scale bar: 500 μm.

As previously described, the *ScDlx2* gene continues to be expressed in the basal telencephalon of embryos at stage 32 (Quintana-Urzainqui et al., 2012). Such expression is high in the *area superficialis basalis* at rostral levels (see Figures 4a–c of Quintana-Urzainqui et al., 2015 and Figure 5b of Docampo-Seara et al., 2020) but at caudal levels, the *ScDlx2* expression is weaker in the *area superficialis basalis* than in neighboring territories (Figure 7a; see also Figure 4e of Quintana-Urzainqui et al., 2015, and Figures 7k,l of Rodríguez-Moldes et al., 2022), which matches the pattern observed with GAD-ir structures (compare with Figure 6d).

The pattern of expression of the *ScNkx2.1* gene in stage-32 embryos reveals the complete absence of labeling in the territory of the *area superficialis basalis* (Figure 7b), which can be unequivocally identified in this stage as shown in Figure 6. However, *ScNkx2.1*-expressing cells can be recognized in the primordial *area centralis subpallialis* and *area lateralis subpallialis* (Figure 7b).

Interestingly, a close observation of the pattern of expression of the pallial marker *ScLhx9* in stage 32-embryos reveals that, in addition to the strong expression in the medial pallium (Quintana-Urzainqui et al., 2015) and in the ventral pallium (Rodríguez-Moldes et al., 2022), a stream of *ScLhx9*-expressing cells, likely originating from the ventral pallium, occupies the marginal neuropile external to the *area superficialis basalis* (Figure 7c, arrowheads). This region and the other subpallial territories lack *ScLhx9* expression (Figure 7c).

It has been previously reported that Pax6-ir cells are absent in the *area superficialis basalis* but form a conspicuous cell group of densely packed Pax6-ir cells around its lateral pole (Rodríguez-Moldes et al., 2022), corresponding to the *area lateralis subpallialis* in the present study. Here, we additionally observe that the absence of Pax6-ir cells in the *area superficialis basalis* contrasts with the relative abundance of these cells in the *area centralis subpallialis*. Moreover, a stream of Pax6-ir cells extends in the marginal neuropile external to the *area superficialis basalis* (Figure 7d, arrowheads), in a similar position to that of *ScLhx9*-expressing cells described above (compare with Figure 7c).

## 4 Discussion

### 4.1 Subdivisions of the Chondrichthyan telencephalon: defining the subpallial territory

To date, there has not been no consensus on the extension of the Chondrichthyan subpallium.

In his extended study of 1978, Northcut defined the Chondrichthyan telencephalon as subdivided into paired evaginated cerebral hemispheres and a more caudal telencephalon medium, formed from the part of the embryonic forebrain that does not expand laterally into the evaginated hemispheres. Northcutt has considered the subpallium as the telencephalic floor of cerebral hemispheres and telencephalon medium and the pallium as the roof of the cerebral hemispheres (Northcutt, 1977, 1978, 1989; Northcutt et al., 1988).

In the atlas of Smeets et al. (1983), which contains a systematic survey of the neuromorphology of the central nervous system of cartilaginous fishes, and in the chapter about cartilaginous fishes in the reference book of Nieuwenhuys et al. (Smeets, 1998), the telencephalon is divided into three parts from rostral to caudal: the *bulbus olfactorius*, the telencephalic hemisphere (subdivided into a dorsal pallium and a ventral subpallium), and the non-evaginated (impar) telencephalon, defined as the portion of the telencephalon that encloses an unpaired ventricle. These authors did not consider the impar telencephalon as part of the subpallium, but rather as a distinct telencephalic subdivision. However, they recognized that some subpallial cell masses (as the *area superficialis basalis*) extended beyond the caudal boundary of the telencephalic hemisphere, which they established at the foramen of Monroi. Some chemoarchitectonic studies in adults of the catshark *S. canicula* (formerly named as lesser spotted dogfish) followed Smeets et al. (1983) telencephalic subdivisions and, thus, considered the impar telencephalon a non-subpallial territory (Rodríguez-Moldes et al., 1990; Molist et al., 1995) while others considered the impar telencephalon to be a subpallial region (Teijido et al., 2002).

Developmental studies have contributed to a better definition of the Chondrichthyan subpallial territory. Such studies, primarily performed in the catshark, have shown that the basic telencephalic territories and genes involved in their specification are conserved in gnathostomes, which makes it possible to definitively identify the extension of the embryonic subpallial territory (Quintana-Urzainqui et al., 2012; Quintana-Urzainqui et al., 2015; Rodríguez-Moldes et al., 2017; Rodríguez-Moldes et al., 2022). Due to the subpallium is the only telencephalic territory where GABA-expressing cells are found in the early catshark embryos, it has been possible to show that the earliest subpallial territory extends in the telencephalic hemispheres and, as Northcutt had considered, in the basal (impar) telencephalon (Ferreiro-Galve et al., 2008; Carrera et al., 2008a; Rodríguez-Moldes, 2009; Quintana-Urzainqui et al., 2012). Moreover, the expression pattern of the *Dlx2* gene (that codifies a transcription factor responsible for the specification of the GABAergic subpallial cell types, and a well-known subpallial marker in embryos of other vertebrates) matches with that of GABAergic cells (Quintana-Urzainqui et al., 2012; Quintana-Urzainqui et al., 2015; Santos-Durán et al., 2022). Thus, in catshark embryos, the telencephalic domain containing GABAergic cells and Dlx2-expressing cells represents the subpallial territory. This evidence, interpreted under the paradigm of the prosomeric model, has revealed that the adult organization of the catshark telencephalon derives from a histogenetic territory that also contains the preoptic area (POA), a subpallial derivative at the non-evaginated part of the telencephalon, i.e., the Northcutt’s caudal telencephalon medium and the Smeets’ impar telencephalon (Carrera et al., 2012; Anadón et al., 2013; Quintana-Urzainqui et al., 2012; Santos-Durán et al., 2015; Santos-Durán et al., 2016; Rodríguez-Moldes et al., 2022; Santos-Durán et al., 2022).

### 4.2 Subdivisions of the Chondrichthyan subpallium

The nomenclature currently used for the cell masses of the Chondrichthyan subpallium (including those of the impar telencephalon) is that of the ones discussed in the studies by Smeets et al. (1983) and Smeets (1998). They are basically the following: *area superficialis basalis*, *area periventricularis ventrolateralis*, Smeets’ striatum, and septal nuclei in the subpallium derived from the evaginated telencephalon; and interstitial nucleus of the basal forebrain bundle, interstitial nucleus of the anterior commissure, entopeduncular nucleus, and preoptic area at the subpallium derived from the non-evaginated telencephalon (impar telencephalon or telencephalon medium). Other cell populations have also been recognized later in the subpallium derived from the evaginated telencephalon: the *area centralis subpallialis* in the catshark *Scyliorhinus* (Manso and Anadón, 1993), probably related to the *area centralis basalis* described by Hofmann and Northcutt (2008) in the ray *Platyrhynoidis*; and the unnamed zone that caps laterally the *area superficialis basalis,* adjacent to the ventral pallium (Rodríguez-Moldes et al., 2022), named here *area lateralis subpallialis*.

Northcutt (1977, 1978) has recognized the *area superficialis basalis* as the *main* zone of the shark subpallium. We agree with this consideration for several reasons. First, it is the more extended and conspicuous subpallial cell population; such conspicuousness is rather exceptional in Chondrichthyans, as it has been noted that the extensive cell migrations and the loss of distinct cell-free zones, especially in telencephalic areas, is a handicap to find their homologies with other vertebrates (Northcutt, 1978; Butler and Hodos, 2005). Second, it is closely related to contiguous cell groups that, despite presenting different cell arrangements, share certain cytoarchitectural, neurochemical, or hodological characteristics with them.

We hypothesize that the *area superficialis basalis* and surrounding territories constitute a complex within the basal subpallium, which we name as the basal subpallial complex. This hypothesis is based on the shared characteristics and unique features of each territory, as we discuss below. The organization of this nuclear complex may reflect the ancestral cell organization from which some subpallial derivatives of jawed vertebrates originally evolved.

### 4.3 Characteristics of the components of the basal subpallial complex

#### 4.3.1 Cell diversity and regionalization of the *area superficialis basalis,* the core of a basal subpallial complex

In the catshark, six neuron types have been distinguished in this area using Golgi methods (Manso and Anadón, 1993), which is described as the subpallial region that contains the highest number of cell types. The most representative described were the large radially oriented bipolar neurons with dendrites extending both superficially and deeply, which form the characteristic dense layer parallel to the surface throughout the entire *area superficialis basalis*. Our data also demonstrate the cellular diversity of the *area superficialis basalis* by showing different types of TH-ir cells, including large bipolar cells and small, round, weakly labeled cells. Some of the large bipolar TH-ir cells with long immunoreactive dendrites that we have observed could correspond to the characteristic large bipolar neurons. These are the only type of TH-ir neurons described in the *area superficialis basalis* of *Squalus* (Northcutt et al., 1988). The smaller, more abundant TH-ir cells described in this study may correspond to the smaller bipolar, stellate, and triangular neurons also described by Golgi methods in the catshark *area superficialis basalis* (Manso and Anadón, 1993). In *Platyrhinoidis* and *Heterodontus,* small round cells were the only type of TH-ir cells described in this area (Stuesse et al., 1990; Stuesse et al., 1994).

**Figure 8:**
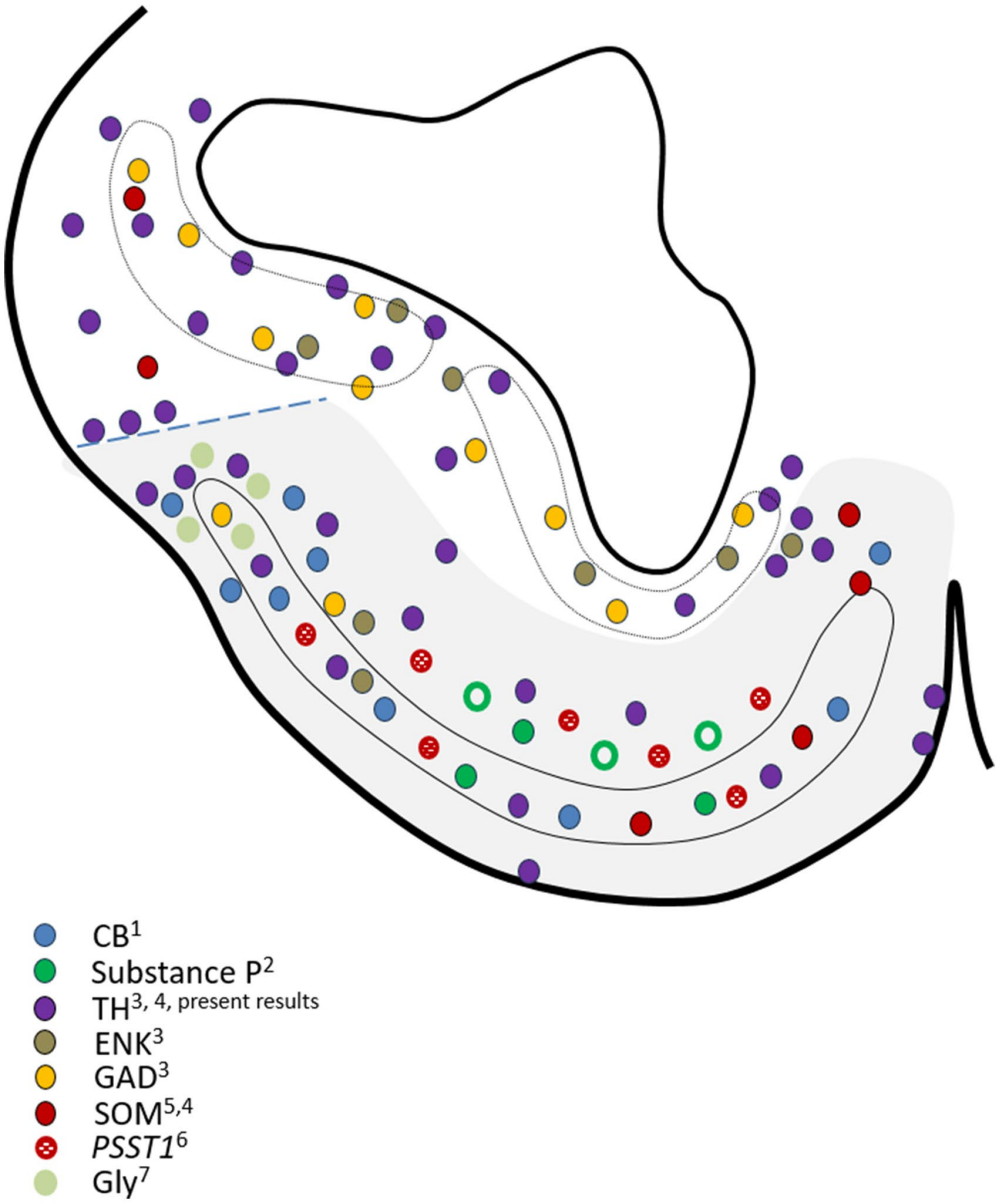
Drawing of a representative transverse section of the adult subpallium of the catshark at an intermediate level used to compile the data available in the Chondrichthyan literature on the chemoarchitecture of the basal subpallium, formed by the *area superficialis basalis* and adjacent territories (gray-shaded area). Source of the data: ^1^Rodríguez-Moldes et al. (1990); ^2^Rodríguez-Moldes et al. (1993); ^3^Quintana-Urzainqui et al. (2012); ^4^Rodríguez-Moldes et al. (2022); ^5^Chiba et al. (1989); ^6^Sobrido-Cameán et al. (2020); ^7^Anadón et al. (2013). Dotted line indicates the presumed pallial–subpallial boundary. For identification of the regions, see Figure 2.

The cellular diversity of the *area superficialis basalis* is also remarkable when considering the variety of cell types based on their neurochemical content. Thus, in addition to the TH-positive cells mentioned above, it contains, in greater or lesser abundance, GABAergic (GAD) cells, glycinergic (Gly) cells, cells containing calcium-binding proteins as calbindin (CB) and calretinin (CR), and neurons expressing a diversity of neuropeptides as Enkephalin (ENK), Substance P, somatostatin (SOM) and the SOM precursor gene *PSST1*, LANT-6, a neurotensin-related hexapeptide, and atrial natriuretic factor or ANF (Reiner and Carraway, 1985; Chiba et al., 1989; Vallarino et al., 1990; Rodríguez-Moldes et al., 1990, 1993, 2022; Quintana-Urzainqui et al., 2012; Sobrido-Cameán et al., 2020; Anadón et al., 2013; present results). In Figure 8, we present the outcome of mapping the information from these articles’ illustrations onto a drawing that represents a mid-level section of the catshark subpallium. The information about LANT-6 and ANF has not been included in Figure 8 because no illustrations of their subpallial distribution were provided in the articles of Reiner and Carraway (1985) and Vallarino et al. (1990), respectively. The neurotensin-related peptide LANT-6 has been shown in numerous cells in the *area superficialis basalis* and near such a cell plate in *Squalus* (Reiner and Carraway, 1985). The peptide ANF was not observed in any subpallial region except the *area superficialis basalis*, where these authors described different types of ANF-containing cells: numerous large and oval perikarya located in the anterior part, and other somewhat smaller perikarya located at more caudal levels (Vallarino et al., 1990). It is interesting to note that cholinergic cells, which were observed in the subpallium of all fish studied using specific antibodies against choline acetyl-transferase (ChAT, the synthetic enzyme for acetylcholine), are absent in the catshark, which has been interpreted as a secondary loss (Rodríguez-Moldes et al., 2002). The absence of ChAT-ir neurons and fibers in the *area superficialis basalis* of the catshark (Anadón et al., 2000) contrasts with the high acetylcholinesterase (AChE) activity in this area but not in neighboring territories (unpublished observations).

The peptide ANF was only observed in cells of the *area superficialis basalis* (Vallarino et al., 1990) and, interestingly, different types of ANF-containing cells were described by these authors. Their observations of numerous large anteriorly located ANF peptide-containing cells and smaller ones caudally located reveal not only the cell diversity of the *area superficialis basalis* but also a clear regionalization between anterior and caudal levels. Our present observations, which indicate that the highest density of innervation of GAD-ir, ENK-ir, and SP-ir fibers is the caudal part of the *area superficialis basalis*, as shown in Figure 4, also support such regionalization. It is interesting to note that such innervation, characterized by abundant perisomatic and peridendritic immunoreactive boutons surrounding immunonegative cells, is mainly noticed in the part of the *area superficialis basalis* that occupies the impar telencephalon derived from the non-evaginated subpallium (what we consider topographically caudal).

**Figure 9:**
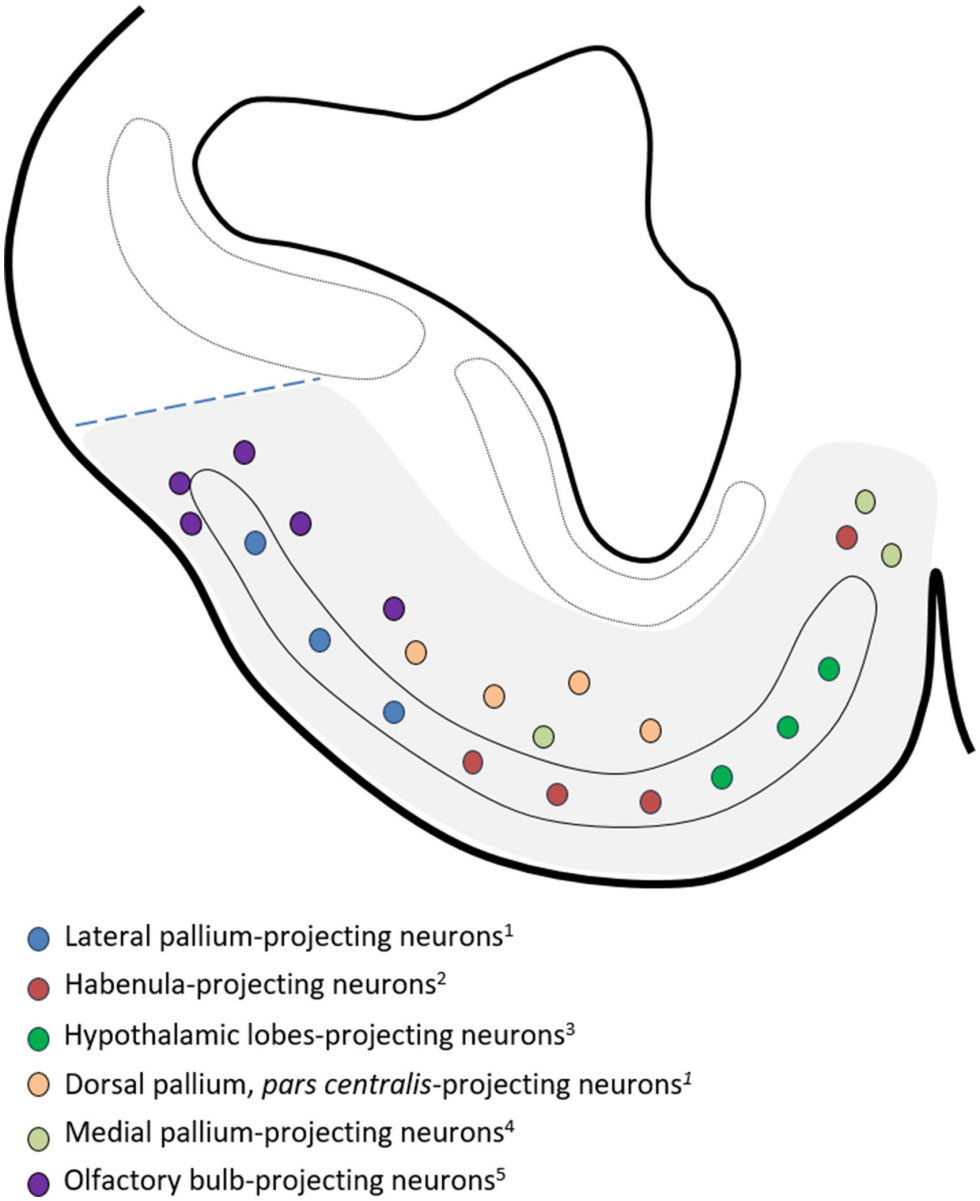
Drawing of a representative transverse section at an intermediate level of the adult subpallium of the catshark used to compile the data available in the Chondrichthyan literature on the location of *area superficialis basalis* projection neurons and adjacent territories, and to show evidence of regionalization of efferences from the basal subpallium (gray-shaded area). Source of the data: ^1^Hofmann and Northcutt (2008); ^2^Giuliani et al. (2002); ^3^Smeets and Boord (1985); ^4^Smeets and Northcutt (1987); ^5^Yáñez et al. (2011); ^6^Smeets (1983). Dotted line indicates the presumed pallial–subpallial boundary. For identification of the regions, see Figure 2.

We have not observed any sign of regionalization anteroposterior or latero-medial in the distribution of TH-ir cells in the *area superficialis basalis* nor has it been noted in other species. The same phenomenon occurs with the distribution of cells containing neuropeptides, except SOM, as a higher density of these cells is observed in the medial part, as illustrated in the article by Chiba et al. (1989), as we have represented in Figure 8. Evidence of the latero-medial regionalization of the *area superficialis basalis* is also supported by the data available regarding its connectivity. We have found evidence of such regionalization after a detailed analysis of results available in the literature referring to the efferent cells of the *area superficialis basalis* in different Chondrichthyan species. In Figure 9, we have compiled such data onto the scheme of a representative transverse section of the adult subpallium of the catshark at an intermediate level. Projection cells were retrogradely labeled in the *area superficialis basalis* after injection of biotinylated dextran amines (BDA) into the lateral pallium of the ray *Platyrhinoidis triseriata* (Hofmann and Northcutt, 2008), DiI into the habenula of the shark *Chiloscyllium arabicum* (Giuliani et al., 2002), and horseradish peroxidase (HRP) into the lateral part of the inferior hypothalamic lobes of the skate *Raja eglanteria* (Smeets and Boord, 1985). Although a possible regionalization of the projections was not explicitly described in the referred studies, their schemata illustrating the distribution of retrogradely labeled cells in the *area superficialis basalis* show that the lateral pallium-projecting cells occupy its mid-lateral part (Figure 4d of Hofmann and Northcutt, 2008); the habenula-projecting cells are at its central part (Figure 3b of Giuliani et al., 2002); and those projecting to the hypothalamic lobes are restricted to its medial part (Figures 7a, 8a–c of Smeets and Boord, 1985). Figure 9 illustrates this interpretation. Another sign of latero-medial regionalization in the *area superficialis basalis* is the density of fibers immunoreactive to neuroactive substances such as calcitonin gene-related peptide (CGRP). No cells immunoreactive to this peptide were observed in the *area superficialis basalis* of the catshark or in any part of the subpallium, except in the entopeduncular nucleus (see below). However, a moderately dense plexus of CGRP-ir varicose fibers has been described, thicker in the central part than in the medial and lateral parts (Molist et al., 1995).

#### 4.3.2 The other components of the basal subpallial complex. Differences and similarities with the *area superficialis basalis*

The detailed analysis of the literature containing data on the *area superficialis basalis* has led us to detect inaccuracies in identifying its extent, which are undoubtedly caused by the similarities between this region and adjacent territories. Thus, the *area superficialis basalis* of Chondrichthyans has been reported as the termination site of fibers with origin in pallium (dorsal pallium and lateral pallium, Hofmann and Northcutt, 2008), and thalamus (Schroeder and Ebbesson, 1974), but illustrations in these studies actually show that the target of such fibers is not the proper *area superficialis basalis* but rather its neighboring areas, that is, the marginal neuropile and the *area centralis subpallialis*. The same is true for projections of secondary olfactory fibers. Olfactory fibers terminating in the *area superficialis basalis* have been described in different species through degeneration studies and with different tracers after injections into the olfactory bulb (Ebbesson and Heimer, 1970; Smeets, 1983; Dryer and Graziadei, 1994; Hofmann and Northcutt, 2008; Yáñez et al., 2011). However, it is worth noting that, considering the images provided, these authors demonstrated that the olfactory fibers running along the lateral olfactory tract do not terminate in the a*rea superficialis basalis*, but in the superficial layer of neuropile and in the vicinity of the superficial region to the *area lateralis subpallialis*. Therefore, we believe that a better understanding of the basal subpallial complex requires recognizing both the unique features of its components and the similarities among them.

The Nucleus N was recognized by Smeets et al. (1983) as the superficial cell layer that replaces the *area superficialis basalis* rostrally. The cytoarchitectonic differences observed between these structures include the larger size of the nucleus N’s cells and their more diffuse arrangement with respect to that of the *area superficialis basalis* (Smeets et al., 1983), and the absence of the characteristic large radial cells of the *area superficialis basalis* (Manso and Anadón, 1993). However, the other cell types observed with Golgi methods in the *area superficialis basalis* are also present in the nucleus N (Manso and Anadón, 1993). This cytoarchitectonic similarity may have led Northcutt (1978) not to recognize it as an independent nucleus, but rather to consider its cells as belonging to the *area superficialis basalis*. We agree with this interpretation based on our current results, which show that the nucleus N contains the same two TH-ir cell types as the *area superficialis basalis*, although a less dense TH-ir innervation (see below). According to our current observations, considering the *area superficialis basalis* as a regionalized structure, the N nucleus would represent its most anterior part.

The entopeduncular nucleus (termed ectopeduncular nucleus by Northcutt et al., 1988, to emphasize the lack of homology with the entopeduncular nucleus of other vertebrates) has been recognized in all Chondrichthyans studied as the nucleus that replaced caudally the *area superficialis basalis,* though it presents a different laminar organization, with more scattered cells (Smeets et al., 1983; Northcutt et al., 1988). According to the study by Manso and Anadón (1993) using Golgi methods, this nucleus has large radial cells similar to those in the *area superficialis basalis*, but it is devoid of the other types of neurons present there. Similarly to the *area superficialis basalis*, habenula-projecting cells have been reported in the entopeduncular nucleus of the shark *Chiloscyllium* (Giuliani et al., 2002), and a dense terminal field of fibers from the lateral pallium has been reported in the skate *Platyrhinoidis* (Hofmann and Northcutt, 2008). In addition, olfactory secondary fibers were found to innervate this nucleus (Hofmann and Northcutt, 2008) and run via its superficial areas (Yáñez et al., 2011). However, there are some differences with the *area superficialis basalis* regarding the cellular organization (less compact), the TH-positive structures contained (see below), and the neurochemical content of their cells. Of note, CGRP-ir cells were not seen in the *area superficialis basalis* of the catshark or anywhere in the subpallium except for the entopeduncular nucleus, where a rather numerous population of small and faintly immunoreactive neurons was described (Molist et al., 1995). The entopeduncular nucleus lies lateral to the basal forebrain bundle, which serves as the main communication channel between the subpallium and the more caudal brain regions, mostly formed by fibers that extend to or originate from the striatum and *area superficialis basalis*, as stated by Smeets et al. (1983).

The *area centralis subpallialis* was defined by Manso and Anadón (1993) as the wide neuron- and neuropile-containing area intermediate between the *area superficialis basalis* and the *area periventricularis ventrolateralis*. Similar cell types to the *area superficialis basalis* were described with Golgi methods, except the large bipolar neurons (Manso and Anadón, 1993). This *area centralis subpallialis* has not been defined by Smeets in any of their reference book and chapters (Smeets et al., 1983; Smeets, 1998), and it could correspond to the *area centralis basalis* of *Platyrhynoidis* described by Hofmann and Northcutt (2008) as a large area dorsal to the *area superficialis basalis.* These authors regarded both cell groups as belonging to what they termed the *Area Basalis*, which is in line with our proposal of a basal subpallial complex. Both territories differ in their projections but contain cells with similar neurochemistry (see Figures 8, 9). As pointed above, it is the *area centralis subpallialis* and not the *area superficialis basalis*, the termination site of fibers with origin in pallium (dorsal pallium and lateral pallium, Hofmann and Northcutt, 2008), and thalamus (Schroeder and Ebbesson, 1974) and it is the *area centralis subpallialis*, and not the *area superficialis basalis,* which contains neurons projecting to the dorsal pallium *pars centralis* (Hofmann and Northcutt, 2008) and to the medial pallium (Smeets and Northcutt, 1987; see Figure 9). Instead, there are many similarities in the presence of cells with similar contents (see Figure 8). In addition, in the *area superficialis basalis*, cells and fibers containing ENK, Substance P, GAD, CB, CR, and TH, and cells expressing the SOM precursor gene *PSST1* have been reported in the *area centralis subpallialis* (Northcutt et al., 1988; Rodríguez-Moldes et al., 1990, 1993, 2022; Quintana-Urzainqui et al., 2012; Sobrido-Cameán et al., 2020; and present results). Differences were noted in the density of cells and of fibers with some of these neurochemical contents or others, being the *area centralis subpallialis* the one that presented the most numerous intense PSST1 + cells (together with septal areas), and the higher density of CGRP-ir innervation (Molist et al., 1995; Sobrido-Cameán et al., 2020). Due to such similarities, we interpret the *area superficialis basalis* and *area centralis subpallialis* as the external and internal strata, respectively, of the basal subpallial complex.

The layer of neuropile and fibers with very few cell bodies that separates the *area superficialis basalis* from the ventral surface of the subpallium is known as the basal (marginal) neuropile (Manso and Anadón, 1993). These authors considered this layer to be part of the *area superficialis basalis*, specifically its neuropile. Our current observations support the fact that, although closely related to the *area superficialis basalis*, the marginal neuropile represents a distinct layer, organized concentrically external to it. We have observed differences in the origin and early development of this layer in relation to the expression of specific genes such as *Lhx9* and Pax6 and the early serotoninergic innervation (see below), which are not seen in the *area superficialis basalis*. Differences in the content of TH-positive structures are also noted (see below). In the present study we have shown monopolar TH-ir cells in the periphery of the marginal neuropile, some of which are placed close to blood vessels, which could correspond to the monopolar cells described by Manso and Anadón (1993) at the periphery of the *area superficialis basalis*. This basal neuropile (and not the *area superficialis basalis*) is the site of termination of fibers originating in the pallium and thalamus (as pointed above) since terminal fields were labeled there after tracer injections into the lateral pallium, dorsal pallium, and forebrain bundles of the ray *Platyrhinoidis triseriata* (Hofmann and Northcutt, 2008). Interestingly, secondary olfactory fibers and thalamic fibers were reported to terminate in this neuropile but with a clear segregation: the terminal field of olfactory fibers being only lateral (Smeets, 1983; Hofmann and Northcutt, 2008) and thalamic fibers being only found at central levels (Schroeder and Ebbesson, 1974). Other hodological studies have reported dense innervation of olfactory fibers throughout the lateromedial extent of this neuropile (Yáñez et al., 2011). GABAergic innervation is higher in the marginal neuropile than in the *area superficialis basalis.* In addition to differences, similarities are also observed, as the marginal neuropile also contains cells expressing the SOM precursor gene *PSST1*, especially at caudal levels (Sobrido-Cameán et al., 2020).

In a previous study, we characterized a catshark subpallial cell population closely attached to the mid-lateral border of the *area superficialis basalis,* in a territory related to the pallial–subpallial boundary, with a characteristic chemical signature (Rodríguez-Moldes et al., 2022). This cell group had not been previously recognized in any reference study on Chondrichthyans, so we refer to it as a*rea lateralis subpallialis.* Comparing the position of this cell population with illustrations from the hodologic studies of Smeets (1983) and Yáñez et al. (2011) on olfactory connections in the catshark, it is clear that our *area lateralis subpallialis* is a subpallial olfactory projection recipient region, distinct from the adjacent pallial olfactory projection recipient region described by these authors as lateral pallium. Northcutt et al. (Northcutt, 1978; Northcutt et al., 1988) characterized in *Squalus* an olfactory-related nucleus containing diffusely distributed cells at the dorsolateral border of the *area superficialis basalis* that they named nucleus A and proposed it as a lateral subdivision of the *area superficialis basalis*, although they did not consider it a subpallial nucleus but a pallial one (ventral to the lateral pallium). We have demonstrated that nucleus A and *area lateralis subpallialis* are different yet related, likely forming part of the amygdalar complex (see below). Our results also show that *area lateralis subpallialis* is closely related to *area superficialis basalis*. Although both populations present clearly different cytoarchitecture (i.e., the population of the *area lateralis subpallialis* is clearly bordering the lateral pole of the compact structure that is the *area superficialis basalis*), they share some features such as the presence of glycinergic cells, TH-positive cells, CB-containing cells (see Figure 9), and Pax6-containing cells (present results; Rodríguez-Moldes et al., 2022).

Northcutt et al. (1988) recognized the septum or septal region of *Squalus* as the medial and ventromedial portion of the subpallium that contained cells aggregated in nuclei, although less densely packed than those at the *area superficialis basalis*. In the catshark, Smeets et al. (1983) recognized the small cluster of cells located near the medial part of *area superficialis basalis* as the *nucleus septi medialis pars posterior*, representing the subpallial septum. In contrast, the nucleus with smaller neurons in a more dorsal position within the medial hemispheric wall, referred by these authors as *nucleus septi medialis pars anterior*, could represent the pallial septum. In *Scyliorhinus,* the subpallial area extending into the basomedial portion of the telencephalic lobes and impar telencephalon was termed *regio septalis* or *nucleus septi* by Manso and Anadón (1993). Like the *area superficialis basalis*, this region contains a relatively homogeneous population of small bipolar neurons and some small and large triangular cells and stellate cells, but lacks its characteristic large radial cells. In the *area septalis* subpallial, neurons were observed projecting to the medial pallium similar to those in the *area centralis subpallialis* in *Squalus* (Smeets and Northcutt, 1987), and habenula-projecting cells identical to those in the central part of the *area superficialis basalis* in the shark, *Chiloscyllium* (Giuliani et al., 2002). The *area septalis* has also been identified in the nurse shark, *Ginglimostoma*, as the termination site of ascending fibers from the thalamus and more caudally (Ebbesson and Schroeder, 1971; Schroeder and Ebbesson, 1974) that run through the basal forebrain bundle (Hofmann and Northcutt, 2008). Intratelencephalic projections from the dorsal pallium pars centralis and *nucleus septi medialis* have also been demonstrated (Hofmann and Northcutt, 2008). Cells expressing the SOM precursor gene *PSST1* have been reported not only in the *area superficialis basalis*, marginal neuropile, and *area centralis subpallialis*, as noted above, but also in septal areas, which are one of the subpallial regions with high-intense *PSST1*-expressing cells (Sobrido-Cameán et al., 2020) and with a high density of SOM-ir cells (Chiba et al., 1989; also see Figure 8).

#### 4.3.3 The catecholaminergic system in the basal subpallial complex

The basal subpallial complex has a catecolaminergic system formed, at least, by different types of cells identified by their TH immunoreactivity that, with a low to moderate density, have been found in the catshark in all populations that are part of the complex defined here. No dopaminergic cells were observed in the *area superficialis basalis* of *Raja radiata* using an anti-dopamine antibody (Meredith and Smeets, 1987), but cells immunoreactive to anti-tyrosine hydroxylase (TH) antibodies have been described in this subpallial area in all Chondrichthyan species studied so far (Northcutt et al., 1988; Stuesse et al., 1990, 1994; Carrera et al., 2012; Quintana-Urzainqui et al., 2012; present results) with some differences in density and sizes between species, as noted above. In *Raja,* the absence of dopamine-ir cells in the *area superficialis basalis* contrasts with the presence of these cells in some adjacent territories, such as the nucleus N, entopeduncular nucleus, and in caudal levels of the septal area (Meredith and Smeets, 1987).

Additionally, a cluster of dopamine-ir cells was observed in an unnamed area of *Raja* around the midline, just ventromedial to the *area superficialis basalis*, which could be related to the monopolar TH-ir cells we have described here in the periphery of the marginal neuropile, some of them located near blood vessels. In *Squalus*, among the territories adjacent to the *area superficialis basalis*, TH-ir cells have only been described in the septal area, although with a lower density than in the *area superficialis basalis* (Northcutt et al., 1988). Notably, they identified TH-ir cells in a territory they defined as the *area periventricularis lateralis*. However, we believe it corresponds to the *area centralis subpallialis* due to its proximity to the *area superficialis basalis* (Figure 5c in Northcutt et al., 1988). Although not explicitly described, TH-ir cells are represented in their illustrations in the marginal neuropile of the *area superficialis basalis*.

The basal subpallial complex appears to have a rich dopaminergic innervation as revealed in *Raja* using an antibody against dopamine (Meredith and Smeets, 1987). Based on the abundant dopamine-ir fibers described in its *area superficialis basalis*, this structure should be considered a dopaminoceptive subpallial region. Although dopamine antibodies have not been used in other Chondrichthyan species, the presence of dispersed (*Squalus:* Northcutt et al., 1988) to moderately dense (*Scyliorhinus:* Carrera et al., 2012; present results) TH-ir fibers in the *area superficialis basalis* could be interpreted as indicative, at least in part, of the reception of dopaminergic fibers. However, the possibility that such fibers belong to non-telencephalic adrenergic or noradrenergic cells projecting to this subpallial area must also be considered. In *Squalus,* scattered TH-ir fibers were also described in the marginal neuropile (Northcutt et al., 1988). In *Raja*, dopamine-ir fibers were of low density and extended among the dopamine-ir neurons in the medial portions of the nucleus N (Meredith and Smeets, 1987). In *Raja*, the area internal to the *area superficialis basalis* appeared innervated by a less dense network of dopamine-ir fibers than that of the *area superficialis basalis* (Meredith and Smeets, 1987). This area of *Raja* could correspond to the *area centralis subpallialis*, although these authors tentatively labeled it as striatum following Smeets and Boord (1985). Furthermore, some dopamine-ir fibers were described in the septal area of *Raja* intermingled among the dopamine-ir cell bodies (Meredith and Smeets, 1987). Dense dopamine-ir and TH-ir fibers have also been described in the basal forebrain bundle of *Raja, Squalus*, and *Scyliorhinus* as well as the possible decussation of many of them in the anterior commissure (Meredith and Smeets, 1987; Northcutt et al., 1988; Carrera et al., 2012; present results).

#### 4.3.4 Origin and early development of the basal subpallial complex populations

Cell populations of the basal subpallial complex derive from histogenetic territories established during embryonic development. Developmental studies in the catshark have revealed protrusions in the evaginated and non-evaginated subpallium that may represent the histogenetic territories equivalent to the ganglionic eminences of mammals (Carrera et al., 2008a; Quintana-Urzainqui et al., 2012). These studies have revealed that cartilaginous fishes are the most basal group of vertebrates that presents a histogenetic field fully comparable to the medial ganglionic eminence (MGE) of tetrapods. The medial protrusion of the catshark subpallium with specific expression of *ScNkx2.1* (a classical marker of the MGE and its derivatives in mammals) and *ScDlx2* (a subpallial marker) observed in embryos at stage 31 (Quintana-Urzainqui et al., 2012) and stage 32 (Quintana-Urzainqui et al., 2015) has been identified as the MGE-like territory. The rest of the subpallial territory, which represents the domain with *ScDlx2*-expressing but *ScNkx2.1-*negative domain, was recognized as the LGE-like domain (Quintana-Urzainqui et al., 2012). Taking into account the subpallial genoarchitecture of the stage-31 embryos previously described and the present results on stage-32 showing the geno- and cytoarchitecture of the early *area superficialis basalis* and neighboring territories, we define the catshark subpallium as formed by a LGE-like derived region containing the *area periventricularis ventrolateralis* and the part of the basal subpallial complex that has the *area superficialis basalis* as its central core and its neighboring territories, the *area centralis subpallialis* (internal), the marginal neuropile (external), and the *area lateralis subpallialis* (lateral). We consider that the MGE-like derived region contains the medial part of the basal subpallial complex, formed by the subpallial septum, and that MGE-like derived cells integrate in the interface between the *area superficialis basalis* and the *area centralis subpallialis* (see below). Nucleus N (interpreted as the most rostral part of the *area superficialis basalis*) and entopeduncular nucleus (at its caudal border) could also be related to such complex. However, their embryonic origin could not be determined in the present study.

Our current observations lead us to consider that in the catshark the early differentiation of the components of the basal subpallial complex takes place at stage 32, based on the fact that (a) in embryos at stage 31, the nuclear groups are not yet established because it is the stage in which the standard primary layers (ventricular, intermediate and marginal layers) of the telencephalic walls are completely formed and the topological organization of the majority of neuronal systems is established (Quintana-Urzainqui et al., 2012), but the nuclear groups are not yet established; (b) at stage 32, as shown in Figure 6, the characteristic cytoarchitecture of the *area superficialis basalis* is clearly recognized with hematoxylin–eosin and with different immunomarkers studied; and (c) at stage 32, the expression of developmental genes involved in the subpallium such as those shown in Figure 7 reveals signs of regionalization based on their differential expression in the primordial territories of the basal subpallial complex. Accordingly, we have selected this stage to define the early organization of the *area superficialis basalis* and neighboring territories.

The differential expression at stage 32 of the four developmental genes analyzed here reveals the first signs of identity of these territories. The incipient territory of the *area superficialis basalis* shows expression of the *ScDlx2* gene but is negative for *ScNkx2.1*; the early *area centralis subpalliales* shows expression of *ScDkx2* and *ScNkx2.1*; in the region of the primordial *area lateralis subpallialis*, *ScDlx2*, *ScNkx2.1*, and Pax6 are expressed; and the early marginal neuropile shows *ScLhx9* expression and Pax6-ir cells in its mid-lateral portion, with a distribution suggesting streams of cells extending lateromedially along the periphery of the basal subpallium.

Furthermore, we have confirmed that the organization of some neural systems described in juveniles and adults is already established at stage 32, while others develop later. Among the first to differentiate early is the GABAergic system. GABA/GAD are reliable subpallial markers in early catshark embryos, as GABA-expressing cells are only present in the subpallium until the pallium is colonized by subpallial-derived GABA-expressing neurons that migrate tangentially from stage-28 embryos. From stages 29 to 31, as numerous GABA-expressing cells colonize the pallium, the density of GABA-expressing cells progressively decreases in the subpallium, although their GABAergic innervation increases (Carrera et al., 2008b). In a recent preprint, Quintana-Urzainqui et al. (2025) have deciphered the trajectories of catshark subpallial GABAergic neurons as they migrate throughout the subpallium and to the pallium, identifying their genetic profiles through transcriptomic profiling, trajectory analysis, and tissue mapping at stage 31 and earlier. We highlight that these authors demonstrate not only that the GABAergic neurons migrating to the shark pallium exhibit a LGE-like profile, but also that the early differentiation of subpallial GABAergic neurons takes place through different trajectories; one of them, a LGE-derived trajectory likely related to the striatum and subpallial amygdala, corresponds to part of the prospective *area superficialis basalis*. In the present study, we have shown that most of the differentiation process of subpallial GABAergic cells must take place at stage 32 since the organization of GAD-ir cells and fibers in the basal subpallium of these embryos is essentially similar to that of juveniles and adults, with poor GAD immunoreactivity in the *area superficialis basalis* that contrasts with the dense GAD-ir innervation in the adjacent territories.

The differentiation of CB-containing cells in the basal subpallium is also early since the distribution of these cells in stage-32 embryos is similar to that of juveniles and adults (Rodríguez-Moldes et al., 1990, 2022), with abundant cells in the *area superficialis basalis,* and moderately dense in adjacent territories such as the *area centralis subpallialis, area lateralis subpallialis*, and septum. In contrast, differentiation of CR-containing cells, at least in the *area superficialis basalis*, appears to occur during postembryonic life, perhaps in late juveniles, as can be inferred by the paucity of these cells in the *area superficialis basalis* observed in stage-32 embryos and in juveniles while the density of these cells in adults is moderate (compare Figures 5a,b, 6f with Figures 5c,d).

It has been previously pointed out that the subpallial peptidergic system containing SP of the catshark starts its differentiation at stage 32, when weak SP immunoreactivity was distinguished at caudal subpallial levels, corresponding to the primordial *area superficialis basalis* (Quintana-Urzainqui et al., 2012).

The present study shows that stage 32 is also the embryonic stage where the pattern of serotonergic innervation is established. The study carried out in the catshark by Carrera et al. (2008b) has revealed that 5HT-ir pioneer telencephalic fibers, which probably arise from the serotonergic neurons of the raphe/reticular formation, reach the telencephalon through the dorsolateral subpallial walls in stage-31 embryos (see Figure 7f of Carrera et al., 2008b). As development progresses, this lateral 5HT-ir innervation extends through the outermost walls of the subpallium and, as we have shown in this study, at stage 32 there is a striking contrast between the very scarce 5HT-ir innervation of the incipient *area superficialis basalis* and the high density of 5HT-ir fibers that border it that are recognized as the incipient *area lateralis subpallialis*, marginal neuropile and septal region. This is the same pattern described in the basal subpallium of the adult catshark, where the highest density of 5HT-ir fibers and their terminal fields (boutons) was observed in the marginal neuropile, in the *area lateralis subpallialis* and in the septum, while the *area centralis subpallialis* showed a low to moderate density of 5HT-ir fibers (Carrera et al., 2008b), which is the same innervation pattern described in other Chondrichthyan species (Northcutt et al., 1988; Yamanaka et al., 1990; Stuesse et al., 1990). These subpallial 5HT-ir fibers must come from extratelencephalic areas, since no serotonergic cells have been reported in the telencephalon of Chondrichthyans.

Other neural systems, such as those of TH-containing populations, develop late in the subpallium. In stage-32 embryos, subpallial populations of catecholaminergic cells and innervation are not yet differentiated. However, the diencephalic and mesencephalic catecholaminergic populations related to the basal ganglia (posterior tubercle nucleus and ventrotegmental area/substantia nigra) are recognized at stages 26 and 31, respectively (Carrera et al., 2005, 2012). No TH-ir cells have been reported in the catshark subpallium at any embryonic stage or in posthatching stages. In contrast, TH-ir cells appeared in the pallium of embryos as early as stage 31 (Carrera et al., 2012). The TH-ir innervation develops late in the subpallium, as the first appearance of TH-ir fibers along the subpallium was not observed until prehatching stages (stages 33 and 34). The developmental study of Carrera et al. (2012) provided evidence of catecholaminergic projections to the subpallium, that course through the forebrain via the basal forebrain bundle, with ascending processes arising from the columnar catecholaminergic groups of the basal diencephalon and mesencephalon in early stages of development (posterior tubercle nucleus, ventrotegmental area/substantia nigra) and reaching the subpallium at prehatching stages.

### 4.4 Basal subpallial complex and basal Banglia: possible homologies

The term basal ganglia mainly includes the striatal and pallidal territories, which originate from the lateral and medial ganglionic eminences (LGE and MGE), respectively. As thoroughly revised in Medina et al. (2014), two major divisions are distinguished in the basal ganglia of tetrapods. The dorsal division, mainly related to somatomotor behavior, includes the dorsal striatum (the caudoputamen complex in mammals) and the dorsal pallidum (the globus pallidus in mammals). The ventral subdivision, mainly related to motivation, includes a ventral striatum (nucleus accumbens and other cell groups) and a ventral pallidum. Whether the *area superficialis basalis* is a pallidal or striatal derivative remains unclear.

The *area superficialis basalis* has been largely considered by some (Reiner and Carraway, 1985; Northcutt et al., 1988) as a pallidal derivative homologous of the proper pallidum (dorsal pallidum or globus pallidus) of amniotes based on: (1) the observations in *Squalus* of numerous immunoreactive neurons to LANT6 (Reiner and Carraway, 1985), a neurotensin-like hexapeptide that appears to be a common trait of the globus pallidus of amniotes (Reiner and Carraway, 1987); and (2) the observation of a poor dopaminergic innervation, together with a relatively high content of fibers immunoreactive for substance P and enkephalin (Northcutt et al., 1988). In *S. canicula*, the presence of cells and a dense plexus immunoreactive to substance P (Rodríguez-Moldes et al., 1993), the poor TH-ir innervation, the dense plexus of ENK-ir fibers, and the presence of few GABAergic cells but abundant GABAergic innervation in the *area superficialis basalis* (Quintana-Urzainqui et al., 2012) could support this homology.

However, the presence in the *area superficialis basalis* of neuronal populations, such as TH-ir neurons, which are not found in the globus pallidus of amniotes, has been considered as an argument against such homology (Northcutt et al., 1988; Smeets, 1998). In addition, it has been pointed out that large aspiny GABAergic neurons with a dense mat of woolly fiber terminals containing GABA, SP, and ENK, which end on the aspiny GABAergic neurons, characterize the globus pallidus of mammals and birds (Kuenzel et al., 2011). Although a few GABAergic cells and abundant GABAergic innervation have been described in the catshark *area superficialis basalis* and the adjacent *area centralis subpallialis* (Quintana-Urzainqui et al., 2012), the present study reveals that such characteristic GABAergic innervation, with high density of perisomatic and peridendritic GAD-ir terminals arranged in a characteristic pattern, is mainly found in the caudal part of the *area superficialis basalis*. At this level, perisomatic and peridendritic SP-ir and, in a lesser density, ENK-ir boutons have also been observed (see Figure 4). Having taken into account these observations, we propose that only this caudal part of the *area superficialis basalis*, which occupies the non-evaginated subpallium, would be homologated to the ventral pallidum.

The *area superficialis basalis* has also been proposed as a striatal derivative, homologous to the olfactory tubercle (ventral striatum) of mammals (Holmgren, 1922; Williams, 1973). Several observations from the present study support this homology. In stage 32 embryos, the territory of the *area superficialis basalis*, well recognized at this stage of development (as seen in Figure 6), does not show *ScNkx2.1* expression, but *ScDlx2* expression, characteristic of the striatal territory, is clearly observed (as seen in Figures 7a,b). Additional evidence supporting this homology includes the TH-ir innervation, which is higher in the *area superficialis basalis* than in any adjacent territory of the basal subpallium, as shown in Figure 4, and the abundance of AChE-positive neurons in this area but not in neighboring territories (unpublished observations).

It has also been pointed out that the *area superficialis basalis* may contain neurons homologous to those of the globus pallidus (dorsal pallidum) and the olfactory tubercle (ventral striatum) of mammals (Northcutt et al., 1988). The presence of LANT6-ir neurons in *area superficialis basalis* of *Squalus* (Reiner and Carraway, 1985) is consistent with this interpretation, as both the pallidum and olfactory tubercle contain LANT6 neurons in amniotes. Smeets (1998) also considered that the *area superficialis basalis* of elasmobranchs consisted of striatal and pallidal elements, which had not yet been segregated.

The possibility that cells of the *area superficialis basalis* have mixed origins from both lateral and medial ganglionic eminence homologs has been proposed, based on evidence of cell migratory pathways in the subpallium of late catshark embryos (Quintana-Urzainqui et al., 2015). This study showed that, in addition to *ScNkx2.1* expression in the MGE-like territory (the prospective pallidal territory), an additional population of *ScNkx2.1*-expressing cells was observed occupying the LGE-like territory (the prospective striatal territory). These *ScNkx2.1*-expressing cells have been considered migrating cells that are part of a pallido-striatal stream (Quintana-Urzainqui et al., 2015).

Our present observations, showing the differential expression of *ScNkx2.1* in the primordial territories of the *area superficialis basalis* and the adjacent *area centralis subpallialis* could help better to delineate the pallidal and striatal territories of the catshark. The absence of *ScNkx2.1* expression that we describe in the *area superficialis basalis* contrasts with the *ScNkx2.*1 expression found at the adjacent territory of the *area centralis subpallialis,* which could explain why this *area centralis*, at least its outermost part, has been interpreted as being within the *area superficialis basalis*. Of note, this is the region that contains the most abundant perisomatic and peridendritic innervation of GABA, Substance P, and ENK. For all these reasons, we propose that this *area superficialis basalis*/*area centralis subpallialis* interface could represent a homolog of the (dorsal) pallidum, and the remaining (more external) *area superficialis basalis* would represent a striatal homolog. The integration of striatal and pallidal neurons has been noticed in the medial striatum of birds, particularly in area X, a specialized structure of this striatal subdivision identified in songbirds (Kuenzel et al., 2011).

Moreover, the evidence of tangential migration of neurons from MGE (and other embryonic domains) to the developing striatum, and from LGE (and other embryonic domains) to the developing pallidum in mammals, together with the available data about the globus pallidus (dorsal pallidum) of nonmammals, has led Medina et al. (2014) to propose two possible modes of development and evolution of the globus pallidus. With the data compiled in the present study, we are persuaded that in the catshark a territory equivalent to the globus pallidus (the *area superficialis basalis*/*area centralis subpallialis* interface) could develop by tangential migration of pallidal-derived cells (*ScNkx2.1*-expressing cells from the MGE-like protrusion) into the developing striatal territory (the *ScNkx2.*1 negative LGE-like territory) as proposed to occur in chicken (Medina et al., 2014).

### 4.5 Basal subpallial complex and subpallial amygdala: possible homologies

The *area superficialis basalis* of cartilaginous fish was proposed by Northcutt (1978) as homologous to the basal (subpallial) amygdala of land vertebrates based on data from Ebbesson (1972) stating that it does not receive direct olfactory input, but massive input from the olfactory-dominated lateral pallium. In a previous study, we suggested that the cell population bordering laterally the *area superficialis basalis* (named here *area lateralis subpallialis*) could be part of the subpallial amygdala, which possibly contained migrated cells of ventropallial origin (Rodríguez-Moldes et al., 2022). Our results showing regionalization between the lateral and medial halves of the *area superficialis basalis* could reflect a functional segregation compatible with the existence of subpallial amygdala components organized differently in relation to olfactory and autonomic functions. Hodologic studies analyzed in the framework of the present study reveal that the mid-lateral half of the *area superficialis basalis* and the adjacent territories, mainly those surrounding its lateral pole, are olfactory-recipient areas (Ebbesson and Heimer, 1970; Smeets et al., 1983; Dryer and Graziadei, 1994; Hofmann and Northcutt, 2008; Yáñez et al., 2011) whereas hypothalamic-projecting cells are in the medial half of the *area superficialis basalis* (Smeets and Boord, 1985), as illustrated in our Figure 9.

Notably, these medially located hypothalamic-projecting cells innervate the inferior hypothalamic lobes, which are large structures characteristic of the hypothalamus of cartilaginous fish (and actinopterygians) involved in feeding and aggression-related behavioral responses (Demski, 1977). These lobes represent a major relay center between the telencephalon and brainstem because of their widespread ascending and descending connections (Smeets and Boord, 1985).

Although further studies are clearly needed to better define the component of the chondrichthyan amygdalar complex, the present study advances the possible existence of components of subpallial amygdala subdivisions related to the *area superficialis basalis*, that is, the olfactory amygdala (mid-lateral), perhaps related to the Medial Amygdala, and the autonomic amygdala (mid-medial), possibly related to the Central (extended) Amygdala of chicken (Vicario et al., 2014). In a broader sense, we consider that there is sufficient evidence to affirm that the *area superficialis basalis* is the subpallial territory where the ancestral/basic components of the subpallial amygdala are settled. Future studies should analyze the possible existence in sharks of an extended centromedial amygdala equivalent to that described in tetrapods (García-López et al., 2008; Moreno et al., 2009; Medina and Abellán, 2012; Medina et al., 2014).

In summary, we can conclude that the *area superficialis basalis* and its surrounding territories constitute a subpallial complex with a precise radial dimension. The core of the complex is the *area superficialis basalis*, a regionalized structure with a remarkable diversity in cell types and their neurochemical content. The organization of some neural systems, such as the GABAergic and serotonergic systems, and the system of cells containing CB, is already established in the *area superficialis basalis* and neighboring territories by stage 32. In contrast, others, such as the TH-containing cells and fibers, develop later. Based on the geno- and cytoarchitecture of the basal subpallium of early prehatching embryos (stage 32), we define the basal subpallial complex formed by a LGE-like derived region containing the *area superficialis basalis* as its central core, the *area centralis subpallialis* (internal), the marginal neuropile (external), and the *area lateralis subpallialis* (lateral); and by a MGE-like derived region formed by the subpallial septum, which represents the medial part of the basal subpallial complex. Moreover, MGE-like derived cells integrate in the interface between the *area superficialis basalis* and the *area centralis subpallialis*. The existence of neurons with mixed origins from both homologs of the lateral and medial ganglionic eminence supports the hypothesis that this interface area may represent a pallidal derivative homologous of the proper pallidum (dorsal pallidum or globus pallidus). In contrast, the remaining *area superficialis basalis* might represent a striatal derivative, with homologies to the olfactory tubercle (ventral striatum) of amniotes. The subpallial basal complex integrates structures homologous to the basic components of both the amygdala and basal ganglia of tetrapods. The organization of this nuclear complex may reflect the ancestral cell organization from which some subpallial derivatives of jawed vertebrates originally evolved.

## Data Availability

All data supporting the findings of this study are available within the article. Further inquiries can be directed to the corresponding author.

## Glossary

ac: anterior commissure
ad: adult
ACS: *area centralis subpallialis*
ALS: *area lateralis subpallialis*
apvl: *area periventricularis ventrolateralis*
ASB: *area superficialis basalis*
CB: calbindin D-28 k
CR: calretinin
Di: diencephalon
DP: dorsal pallium
DPc: caudal part of the dorsal pallium
DCX: doublecortin
ENK: Met-Enkephalin
Ent: entopeduncular nucleus
fbt: basal forebrain bundle (or *fasciculus basalis telencephalic*)
GAD: glutamic acid decarboxylase
Gly: glycine
HE: hematoxylin–eosin
5HT: 5-hydroxytryptamine (serotonin)
Hy: hypothalamus
juv: juvenile
MP: medial pallium
MZ: marginal (basal) neuropile of the subpallium
N: nucleus N
OT: optic tectum
Pall amyg: pallial amygdala
POA: preoptic area
*PSST1*: *somatostatin precursor 1 gene*
sept: septum
SOM: somatostatin
SP: substance P
S-Str: striatum of Smeets
TH: tyrosine hydroxylase
VP: ventral pallium
